# Study of supported heteropolyacid catalysts for one-step DME synthesis from CO_2_ and H_2_†

**DOI:** 10.1039/d4ra07964g

**Published:** 2025-01-02

**Authors:** Anne Wesner, Nick Herrmann, Lasse Prawitt, Angela Ortmann, Jakob Albert, Maximilian J. Poller

**Affiliations:** a Institute of Technical and Macromolecular Chemistry, University of Hamburg Bundesstraße 45 Hamburg 20146 Germany maximilian.poller@uni-hamburg.de +49 40 42838 3172

## Abstract

Dimethyl ether (DME) is a versatile molecule, gaining increasing interest as a viable hydrogen and energy storage solution, pivotal for the transitioning from fossil fuels to environmentally friendly and sustainable energy supply. This research explores a novel approach for the direct conversion of CO_2_ to DME in a fixed-bed reactor, combining the Cu/ZnO/Al_2_O_3_ methanol synthesis catalyst with supported heteropolyacids (HPAs). First, various HPAs, both commercially available and custom-synthesized, were immobilized on Montmorillonite K10. Using a wet impregnation procedure an almost ideal mono-layer of HPA on the support was achieved. The catalysts were further evaluated for their efficiency in direct synthesis of DME from CO_2_/H_2_ in combination with the Cu/ZnO/Al_2_O_3_ catalyst. Among the catalysts tested, tungstosilicic acid (HSiW) supported on K10 exhibited the most promising performance, achieving a DME yield (*Y*_DME_) of 7.06% and a molar productivity (*P*_mol_) of 77.84 mol_DME_ mol_HPA_^−1^ h^−1^. In a subsequent step, further tests using HSiW on various support materials identified ZrO_2_ as the most effective support, increasing the molar productivity to 125.44 mol_DME_ mol_HPA_^−1^ h^−1^, while maintaining the DME yield. The results highlight the potential of applying HPA-based catalysts for sustainable DME synthesis directly from CO_2_, emphasizing the critical role of the catalyst support for optimizing catalytic performance.

## Abstract 2

In this study, various heteropolyacid (HPA) catalysts on different support materials were synthesized and tested in combination with a Cu-based methanol synthesis catalyst for the single-step synthesis of dimethyl ether (DME) from CO_2_ and H_2_.

## Graphical abstract

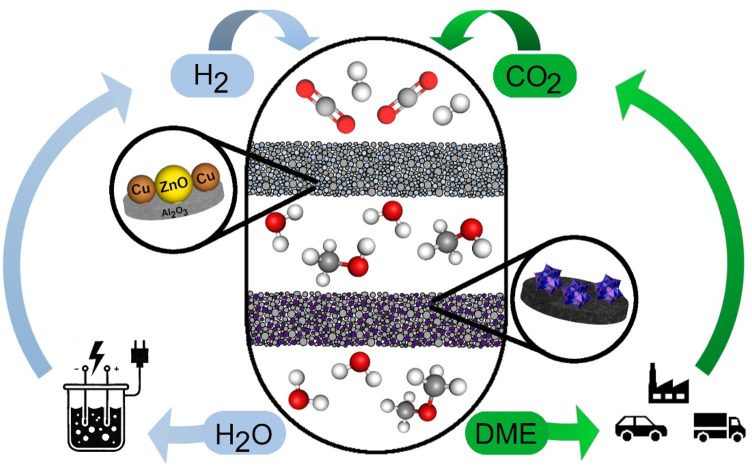

## Introduction

In view of climate change and geopolitical challenges, Europe is turning to renewable energy sources like the sun and wind to reduce dependence on fossil fuels. However, aligning renewable electricity supply with demand is challenging. A viable solution is converting surplus electricity into so-called ‘green’ hydrogen *via* electrolysis, which can then be transformed into methanol (MeOH) or dimethyl ether (DME), effectively storing the hydrogen.^1,2^ DME offers a higher volumetric energy density of 21 MJ L^−1^ compared to hydrogen with 8.5 MJ L^−1^,^3^ is environmentally benign, and easily liquefies under slightly elevated pressure for use with existing liquid gas infrastructure. It already has several applications from propellant to diesel substitute, highlighting its potential as a green energy solution.^4–6^

Typically, DME is produced in a two-step process: first, converting syngas (CO/H_2_) to methanol using a Cu/ZnO/Al_2_O_3_ catalyst, then, in a second step, dehydrating MeOH into DME with a solid acid catalyst.^7,8^ A more efficient approach is the direct synthesis, converting CO or CO_2_ with H_2_ into DME in one step. This method has several advantages, such as simplified operational procedures, increased reaction rates and enhanced equilibrium conversion, achieved through the continuous removal of MeOH as an intermediate from the reaction mixture. Although this process is not yet ready for commercial application, it has gained significant interest from major players in the DME production industry, such as Topsoe, Air Products & Chemicals for its efficiency and potential.^9,10^

The conversion of CO_2_ to DME *via* catalytic hydrogenation is favored from a thermodynamic perspective (eqn (1)). This process requires two different catalytic functionalities: a metallic catalyst for the conversion of CO_2_ to methanol, and a solid acid catalyst for the subsequent dehydration of methanol to DME.^8,11^

1 2CO_2_ + 6H_2_ ↔ CH_3_OCH_3_ + 3H_2_O Δ*H*_298 K_ = −123 kJ mol^−1^

Within the scientific literature, various catalysts with Brønsted or Lewis acidic functionalities have shown to be effective for dehydrating MeOH to DME, with performance depending on the acidic sites' density and strength. Weak and medium acid centers favor DME production, while very strong acid centers may cause formation of other hydrocarbons and coke.^12–14^ Notable catalysts include γ-Al_2_O_3_, H-ZSM-5, mesoporous silicates such as MCM-41 ^15^ or aluminophosphates,^16^ whereby Al_2_O_3_ and H-ZSM-5 are most commonly used.^8,17^ Al_2_O_3_ faces challenges due to the adsorption of water produced during the reaction, which inhibits the active sites.^18^ Conversely, in zeolites like H-ZSM-5, there is a tendency to generate methane or other hydrocarbons as undesirable by-products due to the excessively strong acidic sites.^19^

To overcome the drawbacks of using alumina or zeolites for methanol dehydration, an alternative emerges in the form of Keggin-type heteropolyacids (HPAs) immobilized on supports with high surface areas.^20,21^ These anionic metal-oxide clusters, with the general formula [XM_12_O_40_]^*n*−^, feature a central heteroatom X (typically P or Si) and a metal atom M (usually Mo or W). Their properties can be customized by modifying counterions or metal atoms, tailoring charge, acidity, and pH stability for optimal catalytic performance.^22–24^ Due to their low surface area (approximately 5–10 m^2^ g^−1^), HPAs benefit significantly from being supported on high surface area supports (such as TiO_2_, SiO_2_, ZrO_2_). This approach gains enhanced access to active centers, boosting their activity in methanol dehydration.^6,25–27^

Attributable to their high Brønsted acidity, lacking the excessively strong acidic sites of zeolites, HPAs exhibit remarkable catalytic activity in the dehydration of methanol and have been subject of various studies.^9,12,20,25,28–31^ These studies highlight the strong catalytic performance of HPAs, especially supported H_3_PW_12_O_40_ (HPW) and H_4_SiW_12_O_40_ (HSiW) due to their high acidity.^30,32^ In some instances, these have even outperformed the catalytic activity of H-ZSM-5.^33^ Notably, HPW supported on MCM-41 exhibited a 100% selectivity towards DME from MeOH at equilibrium conversion.^34^ The inherent advantages of HPAs, such as operating under mild conditions, minimizing byproduct formation, thermal stability and resisting deactivation by water, make them especially promising for converting methanol to DME.^9^

To the best of our knowledge, only a limited range of unsubstituted, commercially available HPAs have been utilized in DME synthesis. In this study, the research scope is extended to include transition-metal substituted HPAs to examine the effects of incorporating different heteroatoms such as vanadium and indium. The incorporation of these heteroatoms allow for the modification of the acid sites within the HPAs.^35^ This study aims to explore how varying the acidity through different heteroatoms influences their performance as catalysts in the conversion of methanol to DME. Additionally, this research marks the first instance where both commercial and specially designed catalysts have been evaluated under uniform experimental conditions, enabling a detailed comparative and comprehensive analysis of their catalytic performance. Moreover, diverse supports were employed to further investigate the HPA–support interactions.

## Experimental methods

The following HPAs were supported on Montmorillonite K10 (K10) *via* wet impregnation: H_4_SiW_12_O_40_ (HSiW), H_3_PMo_12_O_40_ (HPMo), H_3_PW_12_O_40_ (HPW), H_8_PV_5_Mo_7_O_40_ (HPVMo), H_6_PInMo_11_O_40_ (HPInMo), and H_4_SiMo_12_O_40_ (HSiMo). Furthermore, HSiW was supported on different carriers (Al_2_O_3_, ZrO_2_ TiO_2_, Celite® 545), using the same method. The supports and catalysts were characterized *via* inductively coupled plasma optical emission spectroscopy (ICP-OES), N_2_-physisorption, X-ray diffraction (XRD), NH_3_-temperature programmed desorption (NH_3_-TPD), scanning electron microscopy (SEM) and infrared spectroscopy (IR). All catalysts were tested in combination with the commercially available Cu/ZnO/Al_2_O_3_ methanol synthesis catalyst in a fixed-bed reactor (Fig. S1†), whereby the two catalyst materials were arranged in two layers separated by a layer of glass wool (Fig. S2†). The reaction conditions were set at 250 °C and 50 bar, with a gas hourly space velocity (GHSV) of 10 000 h^−1^, and a feed gas composition of H_2_/CO_2_ at a ratio of 3 : 1. The gas-phase was analyzed using online gas chromatography (Fig. S3†). An in-depth description of the catalyst synthesis and characterization^35–38^ including all used chemicals (Table S1†), the catalytic experiments^39^ and the catalytic evaluation, can be found in the ESI.†

## Results and discussion

Initially, monolayers of various HPAs, including both commercially available and custom-synthesized variants, were deposited on K10 and their performance was evaluated as part of a bifunctional catalyst system together with commercial Cu/ZnO/Al_2_O_3_ catalyst for DME synthesis. Subsequently, the most promising HPA from the initial screening was combined with different support materials, and their catalytic performance in DME synthesis was systematically evaluated.

### HPA catalyst selection for DME synthesis – supporting of various HPAs on K10

#### Synthesis of various supported HPAs on K10

Initially, various HPAs were immobilized on montmorillonite K10 (K10) as carrier. K10 was chosen as support material based on its previously reported performance, which results from its thermal stability, high surface area, excellent adsorption capacity, and excellent mechanical properties.^12,40^ The acidic properties of K10 can be enhanced through impregnation with HPAs.^41^ The range of HPAs included commercial available HPAs (H_4_SiW_12_O_40_ – HSiW, H_3_PMo_12_O_40_ – HPMo, and H_3_PW_12_O_40_ – HPW) as well as specially synthesized HPAs (H_8_PV_5_Mo_7_O_40_ – HPVMo, H_6_PInMo_11_O_40_ – HPInMo, and H_4_SiMo_12_O_40_ – HSiMo). This selection covers a range of different framework elements (Mo, W), different heteroelements (P, Si), and different charges, resulting in differences concerning the number of protons and their acidic strength.

N_2_ physisorption data reveal that K10, as expected, is a mesoporous layered silicate with an average pore radius just below 2 nm (Table 1). A single Keggin molecule possesses a diameter of approximately 1 nm, indicating that HPA molecules can infiltrate the pores and potentially cover the entire surface area.^35^ The application of HPAs on K10 results in a reduction of the BET surface area by about half in all samples, additionally, a significant decrease in pore volume is also observed. This finding aligns with previous studies, which additionally demonstrated an increase in micropore volume upon impregnation of K10 using HPMo and HPW.^12^

**Table 1:** Textural properties, results of elemental analysis and NH_3_-TPD analysis of supported HPAs on K10

Catalyst	HSiW	HPMo	HPW	HPVMo	HPInMo	HSiMo	Pure K10
**Textural properties**							
*S*_BET_ (m^2^ g^−1^)	97	100	102	112	106	108	215
*Ø* pore diameter (nm)	1.96	1.97	1.97	1.97	1.96	1.96	1.97
Pore volume (mL g^−1^)	0.05	0.09	0.05	0.06	0.10	0.06	0.28

**Elemental analysis**							
W or Mo (wt%)	29.45	20.27	33.53	12.55	23.51	21.50	—
HPA (wt%)	38.42	32.14	43.77	41.15	34.05	30.00	—
Loading_eff_ (μmol_HPA_ g_cat_^−1^)	130	180	150	190	220	190	—
Loading_theor_ (μmol_HPA_ g_cat_^−1^)	160	190	160	200	190	190	—
NH_3_-TPD-normalized adsorption capacity	1.00	1.91	1.02	1.44	2.48	1.36	0.48
Per mass catalyst	1.00	1.91	1.02	1.44	2.48	1.36	0.48
Per molar mass HPA	1.00	1.38	0.88	0.98	1.46	0.93	—

The impregnation of K10 with HPAs aimed at achieving a monolayer of HPA on the entire surface of the support material. The results of elemental analysis (Table 1) were used for the calculation of effective loading (Loading_eff_), which is compared to the maximum theoretical loading (Loading_theor_) to evaluate the impregnation efficiency. Elemental analysis indicates that the impregnation of all HPAs was successful, achieving the target Loading_theor_. For HPMo, HPInMo, and HSiMo, a higher Loading_eff_ is observed, which may be attributed to measurement inaccuracies in the elemental analysis.

**Fig. 1:**
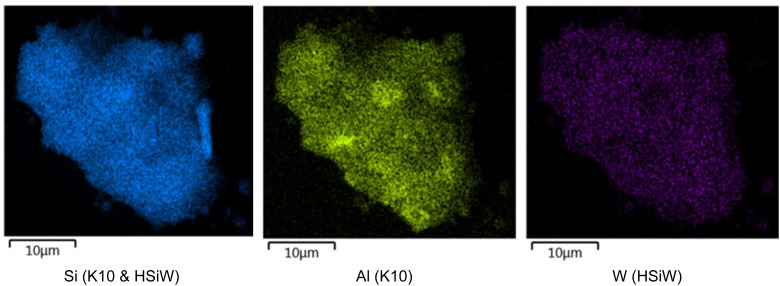
Exemplary SEM EDX-mapping of HSiW supported on K10.

SEM-EDX mapping indicates macroscopic homogeneous distribution of the HPA on the support (Fig. 1 and S4†). Combined with the Loading_eff_ values, which align with the predicted Loading_theor_, this supports the assumption that monolayer coverage has been achieved.

**Fig. 2:**
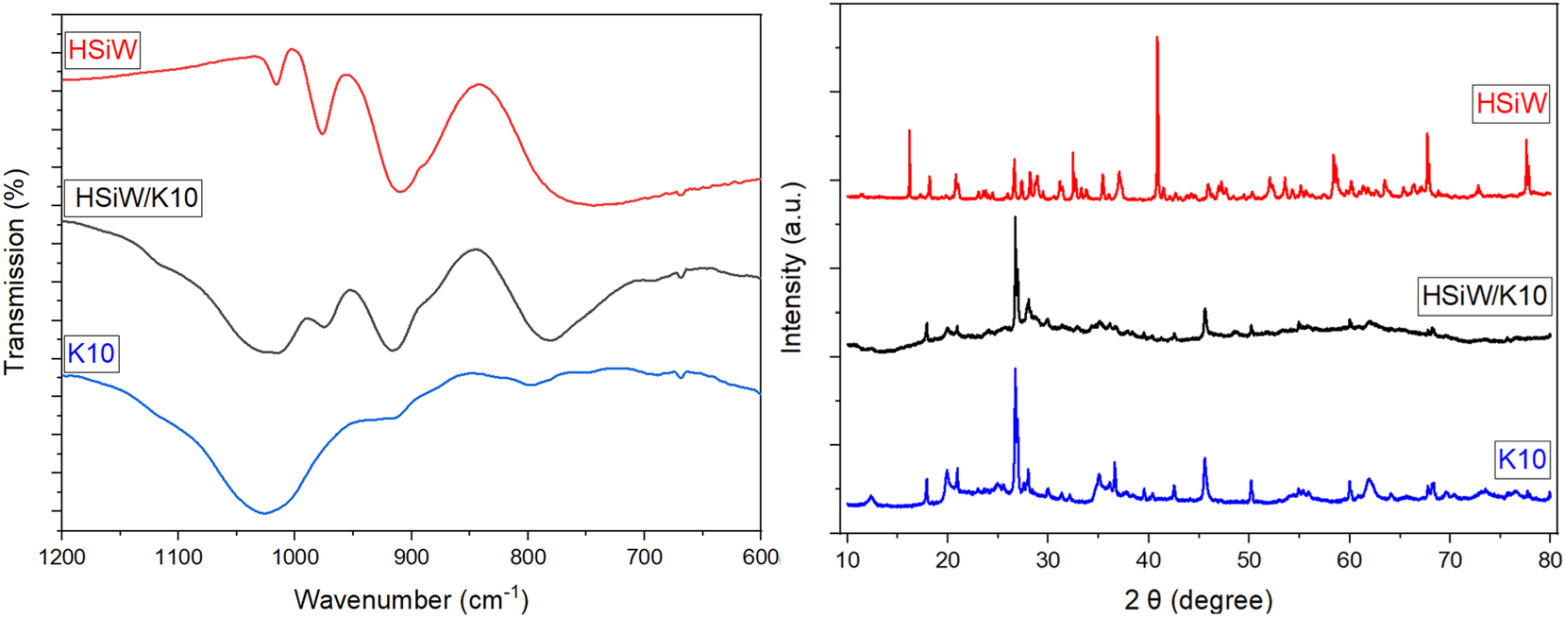
Exemplary IR spectra (left) and XRD (right) of pure HSiW (red line), HSiW supported on K10 (black line) and pure K10 (blue line).

SEM indicates no change in morphology of the catalyst due to the synthesis procedure (Fig. S5†). The preservation of the HPA structure upon supporting on K10 is evident in the IR spectra (Fig. 2 and S6†), apparent by the characteristic Keggin vibration bands: 1049–1060 cm^−1^ for P–O vibration, 945–962 cm^−1^ for M=O_terminal_, 866–877 cm^−1^ for M–O–M_vertex_, and 643–767 cm^−1^ for M–O–M_edge_.^35^ K10 itself displays a very broad vibration band at 1027 cm^−1^ from the stretching vibration of Si–O groups,^42^ which overlaps with the P=O vibration of the HPAs.

Additionally, the samples were characterized by X-ray diffraction (Fig. S7†). It is evident that the characteristic peaks of the support material were preserved after the synthesis, indicating the structure remained intact. However, a reduction in the intensity of the diffraction peaks of pure K10 is observed following impregnation, indicative of a partial loss of crystallinity due to the impregnation process.^41,43^ Furthermore, no peaks corresponding to the HPAs are detected, this is attributed to the insufficient quantity of HPA on the support, resulting in background noise predominance.

**Fig. 3:**
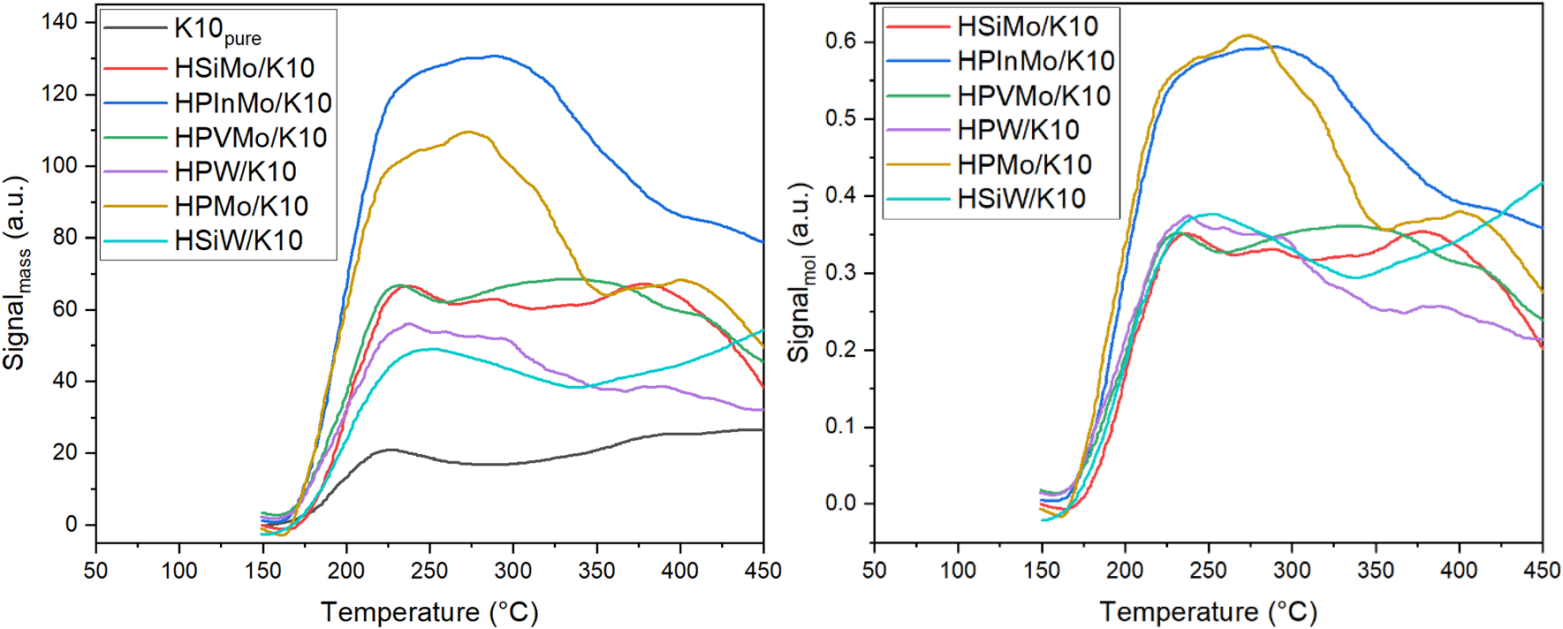
NH_3_-TPD analysis of HPAs supported on K10, normalized to mass of catalyst (left) and normalized to molar mass of supported HPA (right).

NH_3_-TPD data (Table 1 and Fig. 3) indicate varying acidities among the different supported HPAs. It is evident that supporting the HPAs on K10 results in increased acidity compared to pure K10 for all HPAs. The supported catalysts themselves exhibit distinct acidity strengths (Table 1). For instance, HPInMo demonstrates a five-fold higher normalized adsorption capacity of 2.48, related to mass of the catalyst, compared to commercially available HSiW (1.00) and HPW (1.02). The supported, unsubstituted HPMo exhibits a relatively high adsorption capacity of 1.91. In contrast, the incorporation of vanadium (HPVMo) reduces this capacity to 1.44, while HSiMo exhibits an even lower adsorption capacity of 1.36. Thus, incorporation of different heteroatoms allows for targeted adjustment of the acidity of supported HPAs, allowing specific investigation in this study into the impact of acidity on catalytic activity in DME synthesis.

#### Catalytic performance of supported HPAs on K10

**Fig. 4:**
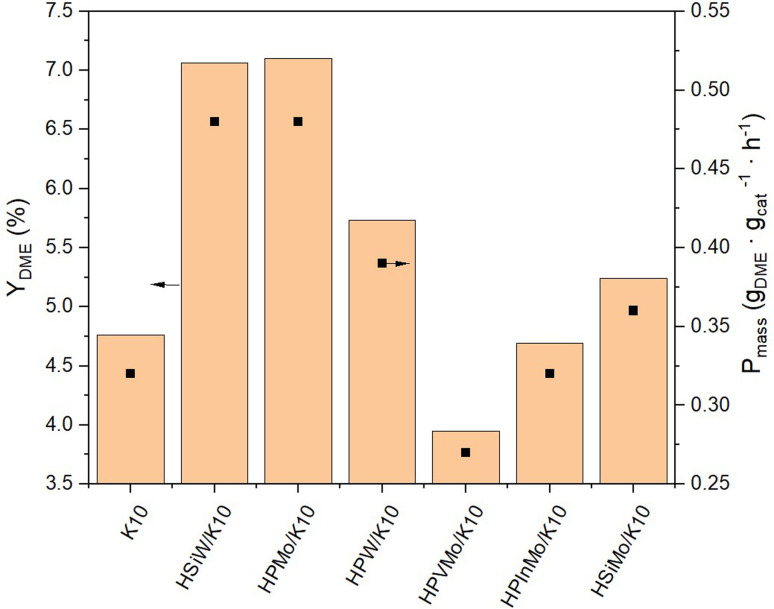
Yield of DME *Y*_DME_ and productivity *P*_mass_ of HPAs supported on K10. Reaction conditions: *T* = 250 °C, *p* = 50 bar, H_2_/CO_2_ 3/1, GHSV = 10 000 h^−1^.

All supported HPAs were tested in combination with the commercial Cu/ZnO/Al_2_O_3_ methanol synthesis catalyst for single-stage DME synthesis from a 3/1 H_2_/CO_2_ mixture (Fig. 4 and Table S2†). Pure K10 already shows a DME yield of 4.76%, resulting from its own acidic sites (Fig. 3 and Table 1). Impregnation with HPInMo and HPVMo results in a decrease in catalytic activity (*Y*_DME_ = 4.69% and 3.95%) compared to pure K10. This reduction in activity could be attributed to the decreased surface area of these HPAs, leading to fewer active sites available on the K10 surface. This limitation could not be compensated by the catalytic efficiency of the HPAs, despite their elevated acidity, which was determined by NH_3_-TPD. Conversely, after impregnation of K10 with HPW and HSiMo, slight increases in catalytic activity were observed, yielding DME of 5.73% and 5.24% respectively, marginally surpassing the performance of pure K10. The highest yields, exceeding 7%, were achieved using HSiW and HPMo impregnated on K10. Under the chosen operating conditions, the thermodynamic DME equilibrium yield of 13%, calculated using the property method Soave–Redlich–Kwong in ASPEN Plus, was not attained using the bifunctional catalyst system, due to the low residence time applied in our setup. The maximum was 54% of equilibrium yield with HPMo/K10 and HSiW/K10.

NH_3_-TPD data (Table 1) reveal no direct correlation between the measured acidity and catalytic activity. For instance, impregnation of K10 with HPInMo increases the acidity fivefold, yet the DME yield decreases post-impregnation compared to pure K10. Conversely, K10 impregnated with HSiW and HPMo, which exhibit the highest catalytic activity, show an acidity increase by just two and four times, respectively, compared to pure K10. This discrepancy can be attributed to the reactions being conducted under optimal conditions for methanol synthesis,^44^ where especially the Brønsted acidic sites of the heteropoly acids have a negligible impact on DME formation.^41^ These conditions were chosen to maximize methanol yield for its subsequent conversion to DME, but leading to no acidity–activity correlation.

The DME selectivities *S*_DME_ for each supported HPA catalyst follow the same trend as for *Y*_DME_ (Fig. S8†). The combined selectivities of DME and MeOH make up approximately 50%, with the remaining 50% attributed to the by-product CO (Table S2†) resulting from the competing reverse water–gas-shift (RWGS) reaction. This indicates that in each experiment conducted, the Cu/ZnO/Al_2_O_3_ catalyst produced almost an equal amount of MeOH and CO, as no further reaction of CO occurs on the DME catalyst.^45^ Consequently, the comparison of DME synthesis activities of the catalysts for the second reaction step is based on consistent conditions.

**Fig. 5:**
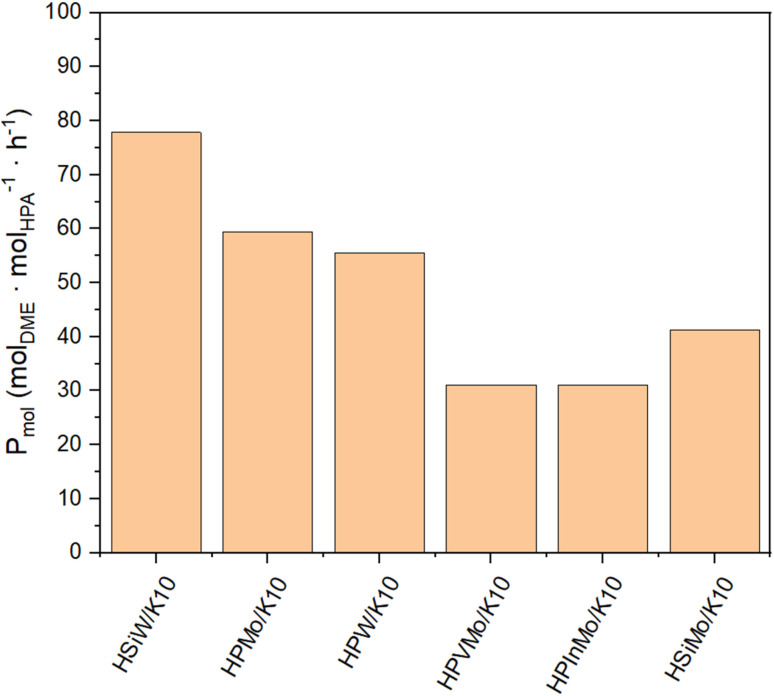
Productivity *P*_mol_ of HPAs supported on K10. Reaction conditions: *T* = 250 °C, *p* = 50 bar, H_2_/CO_2_ 3/1, GHSV = 10 000 h^−1^.

The productivity *P*_mass_ follows the same trend as the DME yield (*Y*_DME_), as a consistent mass of catalyst was used across all synthesis experiments (Fig. 4). However, due to the varying molar masses of the individual HPAs, the molar-based productivity *P*_mol_ shows significant differences (Fig. 5). Here too, HSiW and HPMo on K10 exhibit the highest productivities with 77.84 and 59.40 mol_DME_ mol_HPA_^−1^ h^−1^, respectively, with HSiW/K10 having a higher productivity than HPMo/K10 due to its lower molar mass. HPVMo/K10 and HPInMo/K10 continue to show the lowest *P*_mol_ (both around 30 mol_DME_ mol_HPA_^−1^ h^−1^). The comparison of data between HSiW, HPW, HSiMo, and HPMo on K10 is interesting. Among the tungstates, the Si-containing HPA achieves better results, while HPMo catalyzes the reaction more efficiently than both HSiMo and HPW. Thus, it cannot be stated that either of the metals (W or Mo) offers an advantage, nor is there a trend favoring a central hetero atom (Si or P).

The IR spectra indicate that the Keggin structure is preserved after the reaction across all catalysts (Fig. S9†). The Keggin bands are most distinct for the HSiW/K10 and HPW/K10 catalysts. For all molybdenum-containing HPAs, the vibrational bands are identifiable but exhibit weaker intensity. Additionally, all of the molybdates show a dark blue coloration after the reaction (Fig. S10†), suggesting a reduction process has occurred during the reaction to form molybdenum blue (eqn (2)).^46,47^ The darker coloration and weakening of IR bands indicate that this reduction is incomplete, suggesting the presence of the reduced species of the catalyst as well as poorer catalyst stability.

2 [PMo^VI^_12_O_40_]^3−^ + 4e^−^ ⇌ [PMo^V^_4_Mo^VI^_8_O_40_]^7−^

As an interim conclusion, it is notable that the impregnation of K10 with HSiW and HPMo particularly lead to increased DME yields compared to pure K10. By considering molar-based productivity *P*_mol_, HSiW/K10 is identified as the most efficient catalyst. To validate these findings, the reproducibility of the experimental procedure was investigated using HSiW/K10 in multiple repetitions. These experiments resulted in consistent yields and selectivities for the by-products, MeOH and CO, as well as stable catalyst productivity across the experiments (Fig. S11 and Table S3†), and thereby confirmed the initial results.

### Support selection for DME synthesis – supporting HSiW on different supports

Following the identification of HSiW as the optimal HPA for DME synthesis, its performance was further evaluated on various support materials. To this end, HSiW was immobilized on ZrO_2_, Al_2_O_3_, TiO_2_, and Celite® 545 (hereafter simply referred to as Celite). Celite, primarily composed of SiO_2_, possesses a unique internal structure with vacuoles surrounded by interconnected pores within its silica walls, providing an ideal surface for physical adsorption. Due to its adsorptive and insulating properties, Celite is widely used in applications such as filtration, chromatography, and mild abrasives.^48^ ZrO_2_, Al_2_O_3_, and TiO_2_, on the other hand, are established support materials for supported catalysts, valued for their stability and compatibility with a variety of catalytic processes.^49–52^ The influence of support materials in enhancing the catalytic activity of HPAs for DME synthesis is pivotal, as demonstrated in previous studies, which have highlighted the beneficial effects of utilizing various supports such as SiO_2_ or TiO_2_ for HPAs.^25,53^ However, detailed analyses of the support's influence for HPAs remain insufficiently explored in existing research.

The amount of HSiW used for synthesis was adjusted to the surface area of each support to create a monolayer. The impregnation was carried out as described above. In Table 2 the elemental analysis as well as the effective loading Loading_eff_ and the maximum theoretical loading Loading_theor_ and the point of zero charge of the supports are listed. For all supports, the actual and theoretical loadings closely match, indicating complete impregnation of HSiW on each support. IR spectra confirm the preservation of the Keggin structure of all supported catalysts (Fig. S12†).

**Table 2:** Textural properties and results of elemental analysis of HSiW on different supports

	HSiW/ZrO_2_	ZrO_2_	HSiW/Al_2_O_3_	Al_2_O_3_	HSiW/TiO_2_	TiO_2_	HSiW/Celite	Celite
**Textural properties**								
*S*_BET_ (m^2^ g^−1^)	81	91	161	277	106	163	4	1
*Ø* pore diameter (nm)	3.40	4.07	1.97	4.48	1.86	2.37	1.57	1.85
Pore volume (mL g^−1^)	0.18	0.28	0.23	0.75	0.13	0.33	0.01	0.00
Point of zero charge		6.52		7.6		5.9		7.08

**Elemental analysis**								
W (wt%)	18.32	—	33.24	—	28.91	—	45.02	—
HPA (wt%)	27.19	—	49.34	—	42.91	—	68.81	—
Loading_eff_ (μmol_HPA_ g_cat_^−1^)	80	—	150	—	130	—	210	—
Loading_theor_ (μmol_HPA_ g_cat_^−1^)	90	—	150	—	120	—	210	—

Celite, like K10, represents another silicate used for supporting HSiW. It exhibits a notably low surface area of just 1 m^2^ g^−1^ and no measurable pore volume (Table 2). The minimal surface area measured can be attributed to Celite's very large pores of ≥200 nm, visible in SEM (Fig. S13†). These pores are too large to be quantified using the available BET measurement equipment. Post-impregnation, SEM images indicate pore blockage (Fig. S13†), and the clustering effect increases the measured surface area to 4.35 m^2^ g^−1^.

For the three oxide materials (ZrO_2_, Al_2_O_3_, and TiO_2_), SEM images (Fig. S13†), combined with SEM-EDX images (Fig. S14†), indicate that the particles remain approximately the same size, thus undamaged post-synthesis, and reveal a homogeneous distribution of the HPA across the entire surface. Among these materials, ZrO_2_ has the smallest surface area at 91 m^2^ g^−1^, while Al_2_O_3_ possesses the largest of 277 m^2^ g^−1^. Post-impregnation, the surface areas of Al_2_O_3_ and TiO_2_ decrease by approximately 40%, with a significant reduction in pore volumes as well. Conversely, ZrO_2_ shows only an 11% reduction of surface area, with smaller decreases in pore radius and volume, suggesting a particularly uniform distribution of HPA molecules across the entire surface of the support (Table 2).

**Fig. 6:**
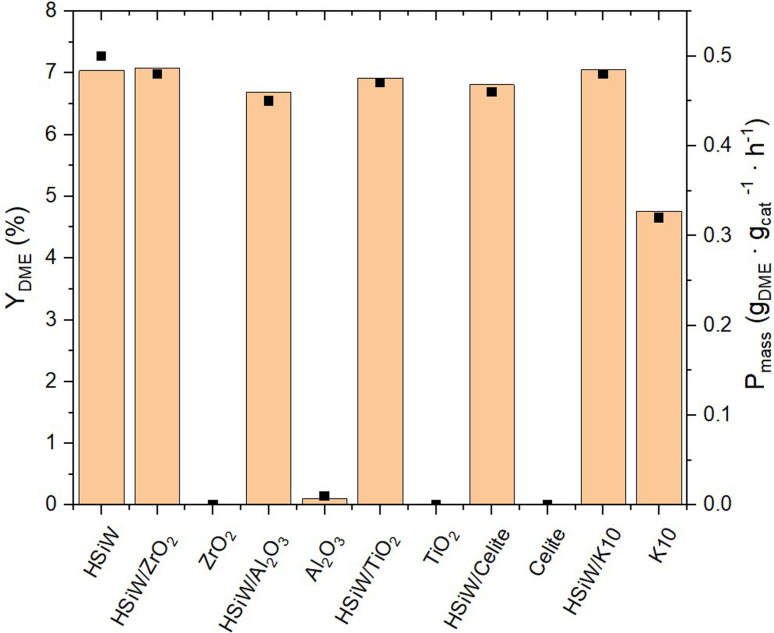
Yield of DME *Y*_DME_ and productivity *P*_mass_ of HSiW on different supports. Reaction conditions: *T* = 250 °C, *p* = 50 bar, H_2_/CO_2_ 3/1, GHSV = 10 000 h^−1^.

The supported catalysts as well as the supports themselves were employed in the synthesis of DME (Fig. 6). Among the tested supports, pure K10 demonstrates significant inherent catalytic activity. The incorporation of HPAs onto the supports invariably lead to an enhanced catalytic performance compared to the unmodified supports. The DME yield across all HPA-modified catalysts is observed to be around 7%, with a *P*_mass_ of 0.5 g_DME_ g_cat_^−1^ h^−1^. Due to the limited precision of the measurements, the productivity data do not decisively distinguish the most effective HPA-support combination. Remarkably, the mass-normalized productivity of unsupported HSiW, matches that of the supported catalyst materials.

**Fig. 7:**
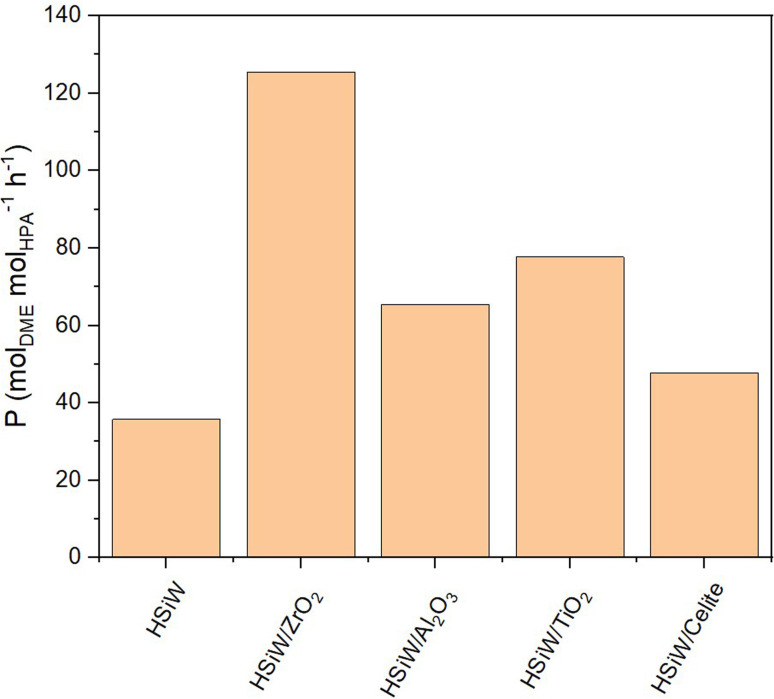
Productivity *P*_mol_ of HSiW on different supports. Reaction conditions: *T* = 250 °C, *p* = 50 bar, H_2_/CO_2_ 3/1, GHSV = 10 000 h^−1^.

When normalizing productivity to the molar amount of catalyst (Fig. 7), unsupported HSiW exhibits the lowest productivity of 35.77 mol_DME_ mol_HPA_^−1^ h^−1^. For each support, it is observed that the catalytic activity is consistently enhanced by the support material. This enhancement is attributed to the generally increased surface area, which improves accessibility to active sites crucial for converting MeOH to DME. Interestingly, catalytic activity does not directly correlate solely with higher surface area and therefore with a higher loading of the HSiW monolayer. Impregnation on Celite slightly increases *P*_mol_ to 47.68 mol_DME_ mol_HPA_^−1^ h^−1^, followed by HSiW on Al_2_O_3_, TiO_2_ and K10, with the HSiW/ZrO_2_ as combination achieving the highest *P*_mol_ of 125.44 mol_DME_ mol_HPA_^−1^ h^−1^. This suggests a cooperative effect between the support and the HPA, which enhances the catalytic activity.

As previously demonstrated and confirmed in this section, the combined selectivities of DME and MeOH consistently make up about 50%, with the remaining 50% attributed to the by-product CO (Fig. S15 and Table S4†). This steady result indicates that MeOH production by Cu/ZnO/Al_2_O_3_ catalyst remains consistent across all experiments, with no further CO conversion by the supported HPA catalyst. This allows for a fair comparison of the DME formation by the supported HPAs in the second reaction step under uniform conditions. The pure supports used for the HPA catalysts showed no catalytic activity for DME synthesis, except for K10, which shows partial conversion of MeOH to DME without any HPA supported.

NH_3_-TPD analysis (Fig. S16†) indicates that catalytic activity also does not directly correlate with measured Brønsted acidity. Specifically, HSiW/ZrO_2_ exhibits the second highest acidity after HSiW/Al_2_O_3_. These findings suggest additional factors influencing catalytic activity beyond surface area and Brønsted acidity. Previous studies indicate that ZrO_2_ provides additional sites for methanol adsorption, enhancing methanol conversion and leading to higher DME production.^25,54^ SEM-EDX analysis and N_2_-physisorption also confirm that despite ZrO_2_'s smaller surface area, it is fully and uniformly covered by HPA after impregnation, ensuring optimal catalytic activity through enhanced accessibility of acid sites, highlighting ZrO_2_ as an exceptional support material.

### Comparative analysis with previously-reported catalyst

The most effective catalyst identified in this study, hereafter referred to as HSiW/ZrO_2_^W^, was compared with the leading literature-reported catalyst for DME synthesis from CO_2_, HSiW/ZrO_2_^K^, as reported by Kubas *et al.*^21^ To enable a direct comparison of the catalytic performance, the catalyst was synthesized following the method outlined by Kubas,^21^ with equivalent HPA-unit loading of 1 HPA unit per nm^2^ of, and subsequently tested under identical reaction conditions.

The catalytic performance (Table 3) of HSiW/ZrO_2_^K^ shows generally good agreement with HSiW/ZrO_2_^W^, with slightly higher values for DME yield (*Y*_DME_ = 7.08%) and selectivity (*S*_DME_ = 30.91%) for HSiW/ZrO_2_^K^, compared to HSiW/ZrO_2_^W^ with *Y*_DME_ = 6.88% and *S*_DME_ = 31.09%. The mass-specific productivities for both catalysts are equivalent, with *P*_mass_ = 0.48 g_DME_ g_cat_^−1^ h^−1^ (HSiW/ZrO_2_^W^) and 0.47 g_DME_ g_cat_^−1^ h^−1^ (HSiW/ZrO_2_^K^). However, due to lower HPA loading, the molar productivity of our HSiW/ZrO_2_^W^ is higher compared to the HSiW/ZrO_2_^K^ catalyst reported by Kubas *et al.*,^21^ indicating a possible improvement in HPA dispersion resulting from the synthesis method we used in this study.

**Table 3:** Catalytic Results for HPA/ZrO_2_ of current study (HPA/ZrO_2_^W^) *vs.* catalyst from literature (HPA/ZrO_2_^K^). Reaction conditions: *T* = 250 °C, *p* = 50 bar, H_2_/CO_2_ 3/1, GHSV = 10 000 h^−1^

Catalyst	HSiW/ZrO_2_^W^	HSiW/ZrO_2_^K^
*X*_CO_2__ (%)	19.36	18.70
*Y*_MeOH_ (%)	3.32	3.40
*Y*_DME_ (%)	7.08	6.88
*Y*_CO_ (%)	12.50	11.85
*S*_MeOH_ (%)	14.50	15.36
*S*_DME_ (%)	30.91	31.09
*S*_CO_ (%)	54.59	53.55
*P*_mass_ (g_DME_ g_cat_^−1^ h^−1^)	0.48	0.47
*P*_mol_ (mol_DME_ mol_HPA_^−1^ h^−1^)	125.44	108.67

Overall, the comparison underscores the enhanced catalytic activity of HSiW supported on ZrO_2_ as a robust support material, irrespective of specific synthesis or reaction conditions. This study further demonstrates, through the use of tailored heteropoly acid catalysts and a range of supports, that parameters such as support surface area, pore size, and the tuned acidity of heteropoly acids do not have a definitive impact on catalytic activity. Notably, HSiW/ZrO_2_ consistently outperforms other polyoxometalates, although the exact underlying mechanisms remain unclear and warrant further investigation.

## Conclusions

In this study, various HPA catalysts were employed for the single-step synthesis of DME. Therefore, bifunctional catalyst systems, combining commercial Cu/ZnO/Al_2_O_3_ catalyst with supported HPAs, have been prepared. Both commercial HPAs (HPW, HPMo, HSiW) and specially synthesized HPAs (HPVMo, HPInMo, HSiMo) were used. The successful impregnation of K10 montmorillonite with monolayers of various HPAs was confirmed by a range of analytical techniques including ICP-OES, SEM-EDX, and N_2_-physisorption. Subsequently, these catalysts were evaluated, in combination with a methanol synthesis catalyst, for their DME synthesis activity in a fixed-bed reactor. HSiW emerged as the most effective catalyst in this screening, achieving a DME yield of 7.06% (53% of the equilibrium yield) and a molar productivity of 77.84 mol_DME_ mol_HPA_^−1^ h^−1^. Upon impregnation onto different supports, HSiW supported on ZrO_2_ proved to be the optimal catalyst, enhancing the molar productivity up to 125.44 mol_DME_ mol_HPA_^−1^ h^−1^. Overall, we evaluated an unprecedented range of heteropolyacids and support materials for this reaction. The results highlight that, beyond the strengths and numbers of acidic centers, the uniform dispersion of HSiW on ZrO_2_ enhances accessibility to catalytic active sites.

## Data availability

The data supporting our article with the title “Study of supported heteropolyacid catalysts for one step DME synthesis from CO_2_ and H_2_” have been included as part of the ESI.† Further information is available on request.

## Author contributions

Anne Wesner was responsible for synthesis and characterization of the catalysts, interpreting data, conceptualizing the experimental workflow, and drafting the manuscript. Nick Herrmann performed supervision and design of catalytic experiments. Lasse Prawitt and Angela Ortmann carried out the catalyst synthesis as well as characterization and conducted all catalytic experiments. Prof. Jakob Albert provided infrastructure and equipment. As principal investigator, Dr Maximilian J. Poller was responsible for conceptualization of this project, acquired financial support, coordinated and supervised the project. All authors contributed to the discussion of the work and the scientific writing.

## Conflicts of interest

There are no conflicts to declare.

## Supplementary Material

RA-015-D4RA07964G-s001
