# Supplementary material for: Study of supported heteropolyacid catalysts for one-step DME synthesis from CO2 and H2
Source: RSC Adv. 2025 Jan 2;15(1):38–47. doi: 10.1039/d4ra07964g (PMC11694141; doi:10.1039/d4ra07964g)
Supplement: RA-015-D4RA07964G-s001 [file RA-015-D4RA07964G-s001.pdf]

Supplementary Information (SI) for RSC Advances.
This journal is © The Royal Society of Chemistry 2024

Study of supported heteropolyacid catalysts for one-step DME synthesis from CO_2 and H_2 – Supplementary Information

Anne Wesner[a#], Nick Herrmann[a], Lasse Prawitt[a], Angela Ortmann[a], Jakob Albert[a], Maximilian J. Poller[a*]

[a]Institute of Technical and Macromolecular Chemistry, University of Hamburg, Bundesstraße 45, 20146 Hamburg, Germany

* Contact details of the corresponding author: Phone: (+49)-40 42838 3172
e-mail: maximilian.poller@uni-hamburg.de

Table of Contents

Catalyst synthesis 2
- Synthesis of HPAs 2
- Supporting of HPAs 4

Catalyst characterization 5

Catalytic evaluation and determination of catalytic parameters 8
- Catalytic experiments 8
- Analysis of reaction products via Gas-Chromatography (GC) 9
- Calculation of catalytic parameters 10

Supplementary results and discussion 13
- HPA catalyst selection for DME synthesis – Supporting of various HPAs on K10 13
- Support selection for DME synthesis - Supporting HSiW on different supports 23

Catalyst synthesis

All chemicals were used as received without further purification for the synthesis of all catalysts.

Table S 1: Chemicals used.

substance	purity / %	company	art.-no.
H_3PO_4	≥ 85	Grüssing	881303334
MoO_3	99.5	Thermo Scientific	206361000
$In(OH)_3$	99.8	Thermo Scientific	011855.18
HCl	n.s.	Thermo Scientific	15401327
$H_4SiW_{12}O_{40}$ x n H_2O	99	Sigma Aldrich	1006590100
Al_2O_3	n.s.	Thermo Scientific	43832
TiO_2	n.s.	Thermo Scientific	44429
Celite® 545	n.s.	Merck	1.02693.0000
Montmorillonite K10	n.s.	Sigma-Aldrich	69866
$Cu/ZnO/Al_2O_3$ catalyst	n.s.	Alfa Aesar	45776
$H_3PMo_{12}O_{40}$	≤ 100	Sigma-Aldrich	79560
$H_3PW_{12}O_{40}$	≤ 100	Sigma-Aldrich	P4006
$NaNO_3$	99	Grüssing	881216623
NaOH	99	Grüssing	881215841

Synthesis of HPAs

Synthesis of $H_6PInMo_{11}O_{40}$ *(HPInMo)*

MoO_3 (20.02 g) and H_3PO_4 (85 %; 1.47 g) were dissolved in water (200 ml) and refluxed with stirring for two hours. During this process, additional H_3PO_4 (0.44 g) was added incrementally. $In(OH)_3$ (2.10 g) was dissolved in 20 ml of water and 10 ml of conc. HCl and then added to the reaction mixture, which was further refluxed for 30 minutes. Subsequently, the solvent was evaporated using a rotary evaporator (80 °C, 200 mbar, 200 rpm), and the product was completely dried (80 °C, 0 mbar, 200 rpm). Synthesis was adapted according *Odyakov*.[1]

Synthesis of $H_8PV_5Mo_7O_{40}$ (HPVMo)

Firstly, MoO_3 (44.3 g) was dispersed in deionized water (500 ml) and mixed with a 25% aqueous H_3PO_4 solution (16.9 g). This mixture was heated to reflux, resulting in a clear yellow solution. Simultaneously, V_2O_5 (20.0 g) was suspended in H_2O (750 ml) and cooled down to 0°C. A 30% H_2O_2-solution (165 ml) was added dropwise while stirring, leading to the dissolution of V_2O_5 into a red/brown solution accompanied by gaseous

O_2 release. After complete dissolution, a 25% aqueous H_3PO_4 (3.0 g) was added and the mixture was stirred at ambient temperature. The V_2O_5-solution was then dropwise combined with the refluxing MoO_3-solution. The mixture was further refluxed for another hour, then allowed to cool to room temperature under reduced pressure, followed by filtration to obtain a red or dark brown solid.[2,3]

Synthesis of $H_4SiMo_{12}O_{40}$ (HSiMo).

MoO_3 (34.55 g) was suspended in 500 ml of H_2O and NaOH (12.75 g) was added until the solid was completely dissolved. Na_2SiO_3 (2.44 g) was solved in a few milliliters of H_2O and added to the molybdate while stirring vigorously. This gave a yellow solution which was acidified with HCl (1 mol/l) to pH 1.4. An unsuccessful attempt was made to extract the product from the acidic reaction solution using diethyl ether. The solution was acidified more to pH 0.745, but the organic phase remained colorless. Organic and aqueous phases were concentrated and 10.00 g of crude product was obtained. The crude product was then dissolved in 100 ml water. A greenish yellow solution was obtained and some white solid settled to the bottom. 10 ml HCl (37 %) and 10 ml H_2O_2 (35 %) were added and the solution turned bright yellow again. Since the white solid did not dissolve again it was filtered off. The filtrate was extracted with 10 x 50 ml $C_4H_8O_2$, causing the organic phase to turn intensely yellow, but the aqueous phase hardly decolorized at all. Lastly, the organic phase was concentrated to dryness and an amorphous green solid was obtained. Synthesis was adapted according Strickland.[4]

Supporting of HPAs

HPA catalyst selection for DME synthesis – Supporting of various HPAs on K10

Montmorillonite K10 was used as a support for various HPAs. For this purpose, 7.01 g of the HPA and 12.00 g of K10 were weighed, to achieve an HPA-unit loading of 1 HPA unit/nm^2. The HPAs that were impregnated included: $H_4SiW_{12}O_{40}$ (HSiW), $H_3PMo_{12}O_{40}$ (HPMo), $H_3PW_{12}O_{40}$ (HPW), $H_8PV_5Mo_7O_{40}$ (HPVMo), $H_6PInMo_{11}O_{40}$ (HPInMo), and $H_4SiMo_{12}O_{40}$ (HSiMo).

The HPA was dissolved in water (500 ml), and used at the resulting pH value without further adjustment, before the support was added. The suspension was mixed for three hours using a rotary evaporator (room temperature, 800 mbar, 111 rpm) and then the solvent was evaporated (80 °C, 200 mbar, 111 rpm). The product was dried for 20 hours at 100 °C.

Support selection for DME synthesis - Supporting HSiW on different supports

Different supports have been used for impregnation of HSiW: Al_2O_3, ZrO_2 TiO_2, Celite® 545. The metal oxides (Al_2O_3, TiO_2, ZrO_2) were initially ground using a mortar to achieve a particle size fraction ranging from 80 to 250 µm. Celite® 545 was used as received without any treatment. The synthesis procedure was analogous to that of different HPAs on K10.

Catalyst characterization

Inductively coupled plasma optical emission spectroscopy (ICP-OES)

The elemental composition of the catalysts was determined using ICP-OES. For microwave digestion, 10 to 20 mg of each catalyst was dissolved in a mixture of reverse aqua regia (HCl/HNO3 1:3) (5 ml) and 1 ml HF, then diluted to 50 ml. The sample was then atomized in an argon plasma. Quantification was based on the relative intensity of the element-specific spectral lines, using previously established calibration curves for the elements of interest. Measurements were carried out by the Central Element Analysis Service of the Department of Chemistry at the University of Hamburg, using an ASCOR spectrometer from Spectro.

N_2-physisorption

N_2-physisorption was performed to determine the textural properties of the materials. Measurements were conducted using an Autosorb iQ MP/XR instrument from Anton Paar, with Quantachrome® ASiQwin™ software utilized for data evaluation. The samples were degassed at 200°C for 10 hours under vacuum to remove surface water. The surface area was determined using the BET (Brunauer-Emmett-Teller) method, while pore volume and average pore diameter were derived from the BJH (Barrett-Joyner-Halenda) desorption isotherms.

X-ray diffraction (XRD)

The crystal structure of powdered catalysts was analyzed using XRD. This was conducted on a Panalytical MDP X’Pert Pro diffractometer, utilizing X-ray radiation generated at a copper source that diffracts off the lattice planes of the crystal samples. The diffraction pattern was recorded by a detector in the range of 10-80° with a scan rate of 0.013° per 0.3 seconds. The resulting diffractogram was processed and evaluated using X´Pert HighScore Plus software.

NH_3-Temperature Programmed Desorption (NH_3-TPD)

The acidity of the catalysts was determined using NH_3-TPD. Measurements were carried out on a Chembet Pulsar TPD/TPR analyzer from Quantachrome Instruments, the data were analyzed using the ASiQwin software. For this analysis, 160 mg of the catalyst was placed in a measurement cell and pre-treated at 150 °C for 1 hour under a helium flow of 80 ml/min to remove any adsorbed species. This was followed by cooling down of the sample to 100 °C and the adsorption of NH_3 at this temperature to saturate the catalyst's surface. Subsequently, loosely bound NH_3 was removed by flowing helium (80 ml/min) over the sample for 1 hour. Finally, TPD measurements were conducted from 150 to 450°C at a heating rate of 10 K/min. The amount of desorbed NH_3 was quantified using a Thermal Conductivity Detector (TCD). The areas under the NH_3-TPD curves, were integrated using Origin software. This analysis normalized all measurements against HSiW/ZrO_2 as standard to ensure consistency and comparability across the samples.

Microscopy

Scanning electron microscopy (SEM) measurements were employed to elucidate the metal dispersion and morphology of the catalysts. These measurements were carried out using a Leo 1550 Gemini scanning electron microscope from Zeiss. For SEM imaging, an acceleration voltage of 2 kV and an aperture diameter of 7.5 µm were utilized. Element distribution maps were created using an acceleration voltage of 20 kV and a 30 µm aperture. The Silicon Drift Detector Ultim Max 100 from Oxford Instruments, combined with AZtec software, was used as the detector. The mapping process for each sample required 10-15 minutes.

Point of zero charge measurement

The determination of the point of zero charge (PZC) for the supports was conducted with a Lab 850 pH meter from Fisher Scientific, using a SI Analytics BlueLine 14 pH electrode for precise pH measurements. To achieve a range of pH values from 2 to 11, adjustments were made in 40 ml of sodium nitrate solution (0.1 M) utilizing NaOH (0.1 M and 0.005 M) and HNO_3 (0.1 M and 0.05 M), thereby setting the initial pH

($pH_{initial}$) for each experiment. A suspension was then prepared by combining each solution with the support material and stirring continuously at 300 rpm for 24 hours. Subsequently, the pH of each solution was measured (pH_{final}). For analysis, the change in pH ($\Delta pH = pH_{final} - pH_{initial}$) was calculated for each suspension and plotted against $pH_{initial}$. The intersection of this line with the x-axis (where $\Delta pH = 0$) was determined to be the PZC of the support material.

Infrared spectroscopy (IR)

IR was utilized for structural elucidation and specifically for the analysis of supported catalysts. Measurements were carried out using an IRSpirit equipped with a QUATR-S unit from Shimadzu, covering a range from 400 to 4000 cm^{-1}.

Catalytic evaluation and determination of catalytic parameters

Catalytic experiments

The experiments were carried out in a stainless steel (1.4571 grade) fixed-bed reactor with an internal diameter of 2 cm, capable of being enclosed by a heating jacket. The flow diagram for the reactor setup, including its peripherals and the connected gas chromatograph (GC), is depicted in Figure S 1.

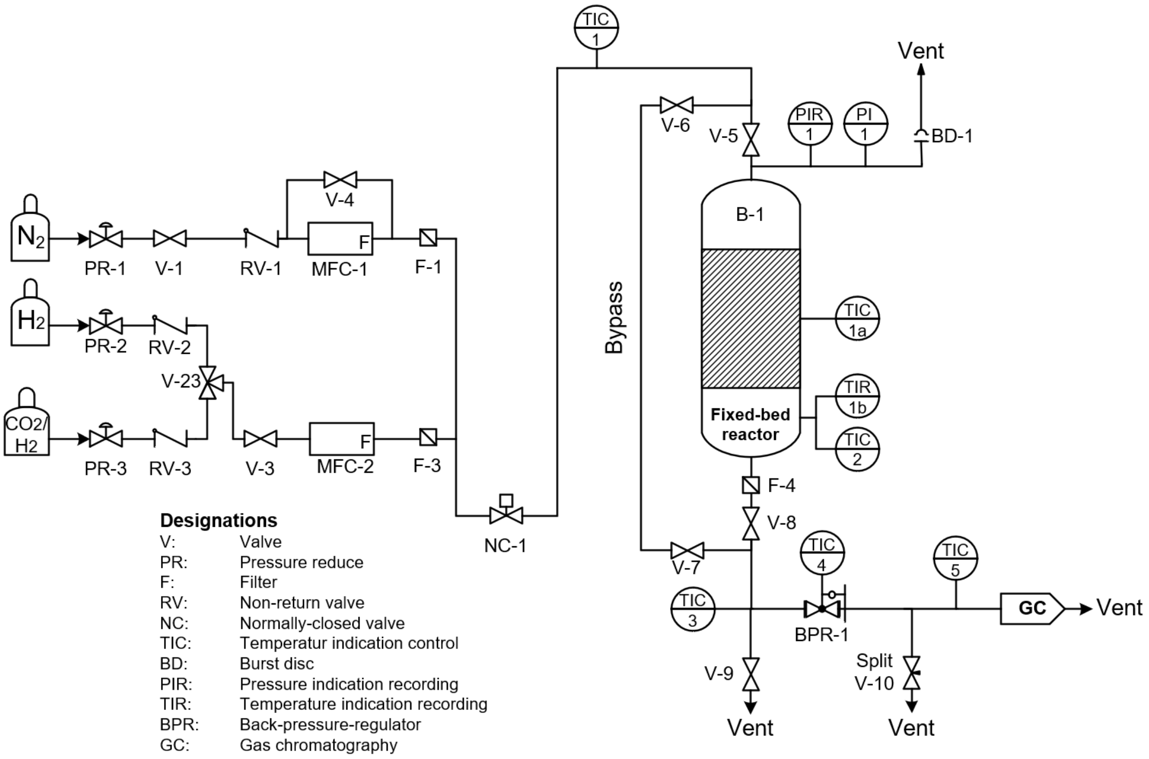

Figure S 1: Flow Diagram of the fixed-bed reactor setup.[5]

For each experiment, 2.5 g of $Cu/ZnO/Al_2O_3$-catalyst and the dehydration catalyst for converting methanol to DME were used. To achieve a bed height of 2 cm for each catalyst, the weighed catalysts were supplemented with inert glass beads (diameter 200-300 µm). Inside the reactor, the two catalysts were separated by a layer of glass wool (2 g). Figure S 2 schematically illustrates the catalyst packing within the reactor, with additional glass wool placed above the methanol catalyst to shape the flow profile and prevent catalyst swirling. The thermocouple (colored orange in Figure S 2) was positioned in the lower part of the methanol catalyst layer.

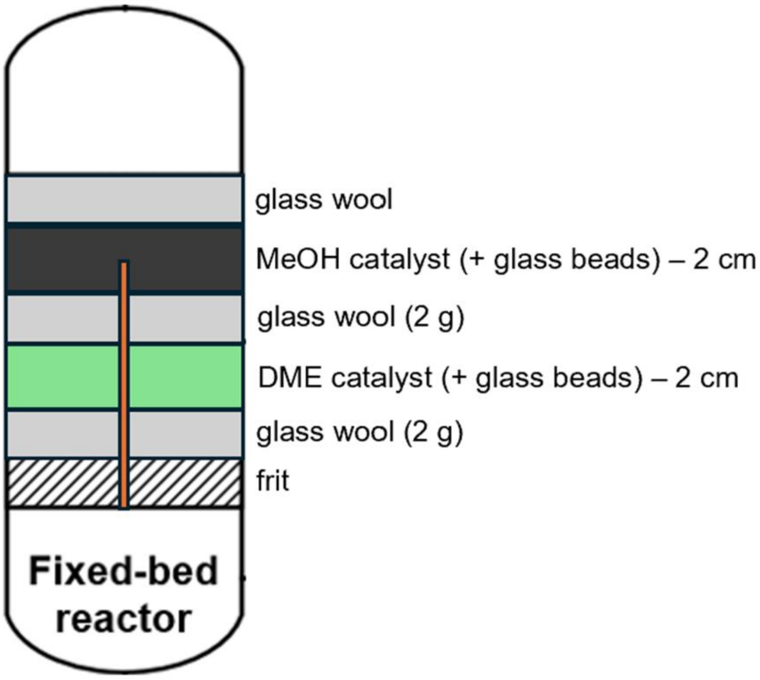

Figure S 2: Schematic Layout of the Catalyst Packing in the Reactor.

The reactor was heated to the reaction temperature of 250 °C under nitrogen flow (300 ml·min^{-1}) with a holding time of 30 minutes. For the one-hour preforming of the methanol catalyst, a gas flow containing 10 % hydrogen was introduced. Subsequently, N_2 was used to establish the reaction pressure of 50 bar. The introduction of the reaction gas (H_2/CO_2 in a 3 : 1 stoichiometric ratio, 1100 ml·min^{-1}) set the start of the reaction. The gas phase was analyzed using online GC after 10, 20, 30, 60, 90, and 120 minutes. To terminate the reaction, the supply of reaction gas was stopped, and the reactor setup was flushed with N_2 and cooled down to room temperature.

Analysis of reaction products via Gas-Chromatography (GC)

The composition of the gas phase at various stages of the reaction was analyzed using an online gas chromatograph Bruker 450-GC from Bruker. A schematic diagram of the GC setup can be seen in Figure S3. The gas was directly transported from the reactor to the GC through a heated gas line. The sample loop was filled with the gas to be analyzed, which was then injected into the column oven with argon as the carrier gas. Four separation columns installed in series and parallel (RT-Q-Bond, RT-U-Bond,

BRSwax, and BR-Molsieve 5A) were used to separate the gas into its components. Detection of MeOH and DME was carried out using the rear flame ionization detector (FID-rear). CO and CO_2 were measured using a methanizer unit at the middle flame ionization detector (FID-middle). Hydrogen was identified using the front thermal conductivity detector (TCD-front).

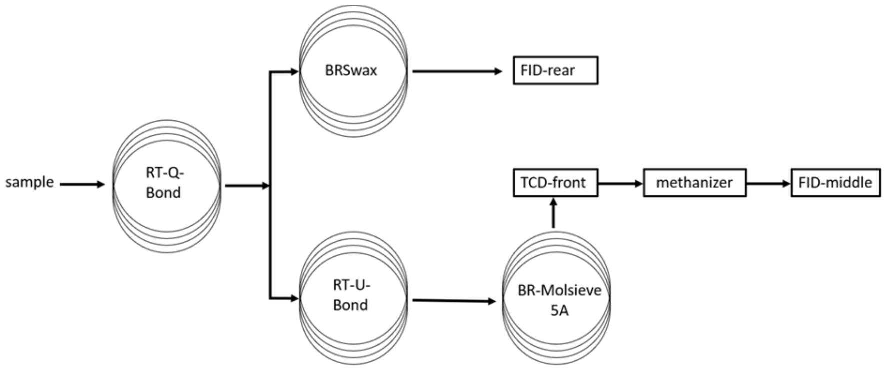

Figure S 3: Schematic Diagram of the Online Gas Chromatograph.

Calculation of catalytic parameters

Using ideal gas law

$$pV=nRT \quad \text{Eq. 1}$$

the molar flow rate $\dot{n}_i$ of a component ii into the reactor can be calculated using equation 2.

$$\dot{n}_{i.in} = \frac{p_N \cdot y_{i.in} \cdot \dot{V}_N}{R \cdot T_N} \quad \text{Eq. 2}$$

Here, p_N and T_N represent the standard pressure and standard temperature, respectively, R is the universal gas constant, $\dot{V}_N$ is the set volume flow rate, and $y_{i.in}$ is the fraction of component i in the input feed gas. The carbon balance of the reaction system is determined by the results of the online gas chromatography, using the average values of the measurements after 20 and 30 minutes for the fractions y_{out} of

the individual components. Firstly, the molar flow of the reactor outlet is calculated with equation 3, followed calculation of molar flow of each component with equation 4,

$$\dot{n}_{ges.out}=\frac{\dot{n}_{CO_2.\ in}}{y_{CO_2.out}+y_{CO.out}+y_{MeOH.out}+y_{DME.out}} \qquad \text{Eq. 3}$$

$$n_{i.out}=\dot{n}_{ges.out}\cdot y_{i.out} \qquad \text{Eq. 4}$$

Thus, yield (Y) and selectivity (S) can be determined using equations 5 and 6:

$$Y_i=\frac{\dot{n}_{i.out}-\dot{n}_{i.in}}{\dot{n}_{CO_2.\ in}}\cdot\frac{|v_{CO_2}|}{v_i}\cdot 100\ \% \qquad \text{Eq. 5}$$

$$S_i=\frac{\dot{n}_{i.out}-\dot{n}_{i.in}}{\dot{n}_{CO_2.\ in}-\dot{n}_{CO_2.out}}\cdot\frac{|v_{CO_2}|}{v_i}\cdot 100\ \% \qquad \text{Eq. 6}$$

The selectivities of the three exclusively detected gases at the outlet were subsequently normalized, due to:

$$S_{CO}+S_{DME}+S_{MeOH}=100\ \% \qquad \text{Eq. 7}$$

The productivity was calculated trough:

$$P_{cat}=\frac{\dot{n}_{DME.out}\cdot M_{DME}}{m_{cat}} \qquad \text{Eq. 8}$$

To enhance the comparability of the HPAs, productivity was additionally calculated relative to the amount of catalyst used:

$$P_n=P_{cat}\cdot\frac{m_{cat}}{M_{DME}\cdot n_{HPA}} \qquad \text{Eq. 9}$$

The CO_2 conversion rate X_{CO_2} and the gas hourly space velocity (GHSV) is determined using:

$$X_{CO_2} = \frac{\dot{n}_{CO_2.in} - \dot{n}_{CO_2.out}}{\dot{n}_{CO_2.\,in}} \cdot 100\ \%$$ Eq. 10

$$GHSV = \frac{\dot{V}_N}{V_{cat}}$$ Eq. 11

Effective molar loading was calculated from the mass fraction of the metal in the catalyst determined by ICP-OES, w_M, the mass fraction of that same metal in the HPA, $w_{M.HPA}$, and the molar mass of the HPA, M_{HPA}.

Eq. 12

$$Loading_{eff} = \frac{w_M}{M_{HPA} \cdot w_{M.HPA}}$$

Theoretical loading was calculated assuming all HPA material used in the synthesis was transferred onto the support.

Eq. 13

$$Loading_{theo} = \frac{m_{HPA}}{(m_{HPA} + m_{support}) \cdot M_{HPA}}$$

The arithmetic mean $\bar{x}$ and the standard deviation σ are determined according to:

$$\bar{x} = \frac{1}{n} \sum_{i=1}^{n} x_i$$ Eq. 14

$$\sigma = \sqrt{\frac{\sum_{i=1}^{n} (x_i - \bar{x})^2}{n-1}}$$ Eq. 15

Whereby *n* is he number of trials and x_i the measured value in the respective trial *i*.

Supplementary results and discussion

HPA catalyst selection for DME synthesis – Supporting of various HPAs on K10

HSiW/K10

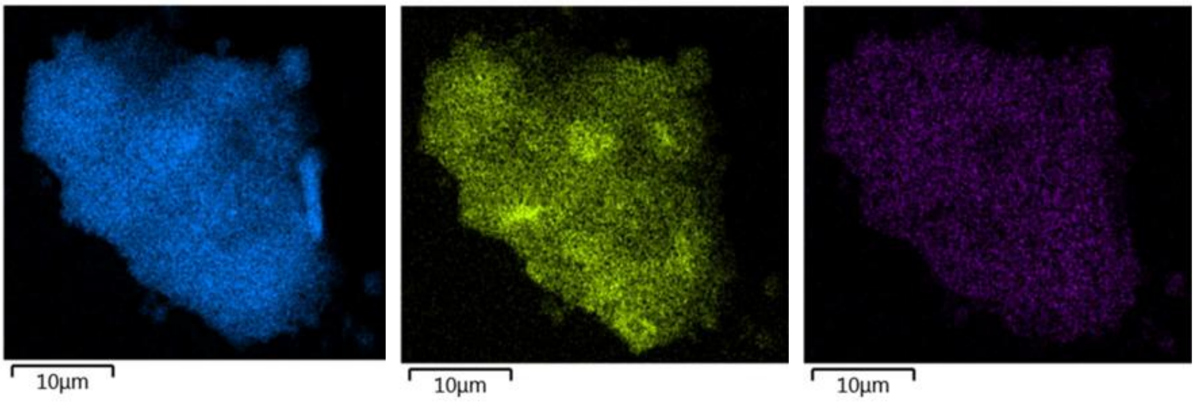

Si (K10 & HSIW) | Al (K10) | W

HPMo/K10

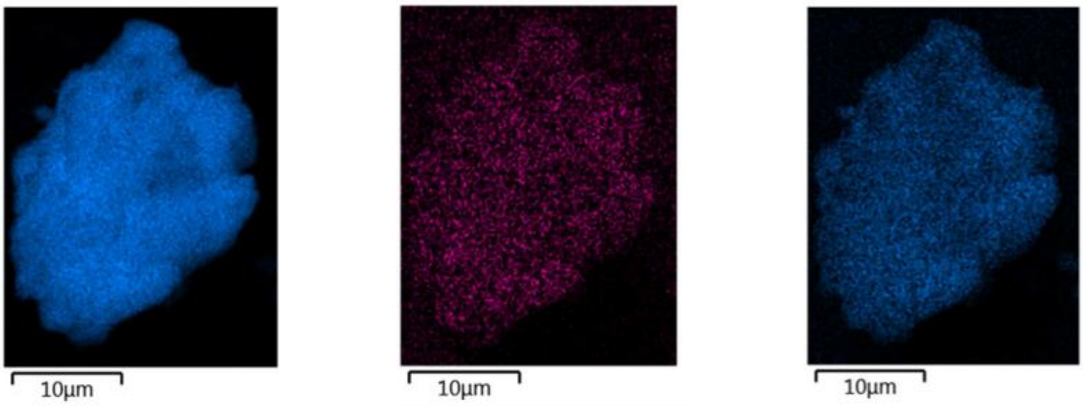

Si (K10) | P | Mo

HPW/K10

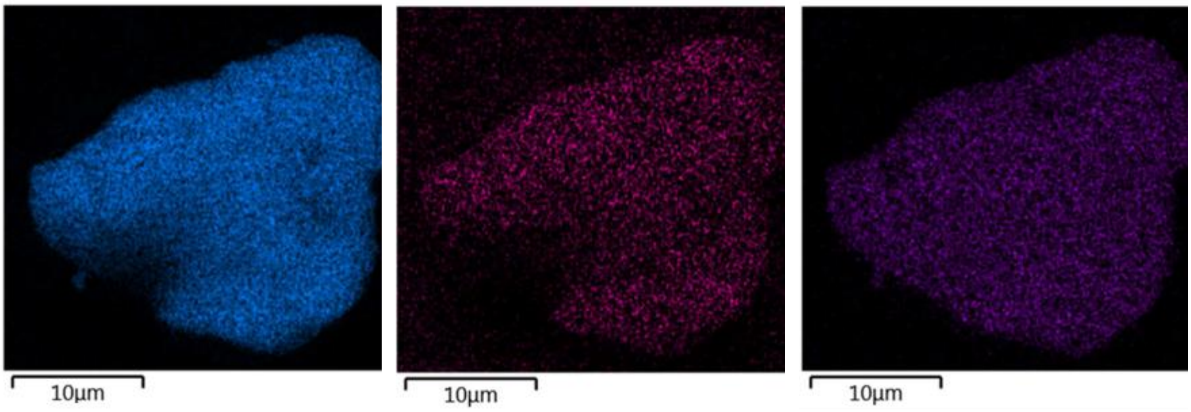

Si (K10) | P | W

HPVMo/K10

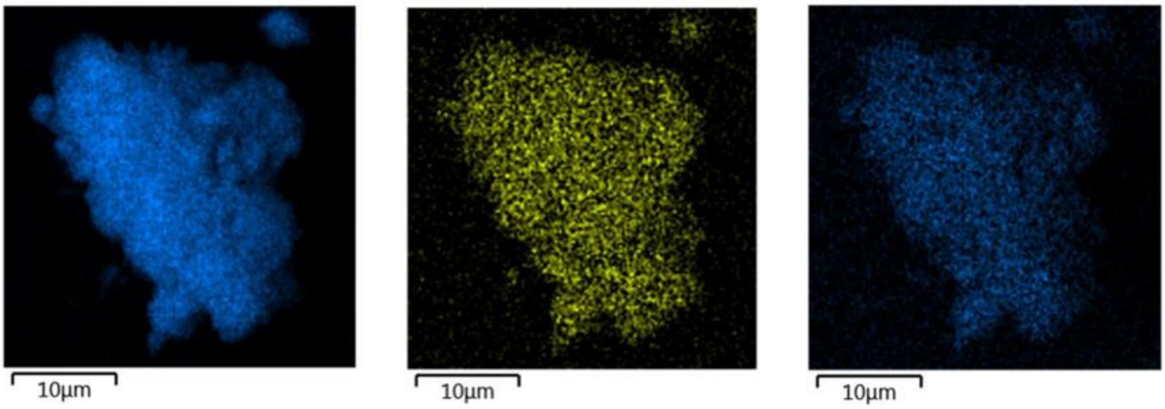

Si (K10) V Mo

HPInMo/K10

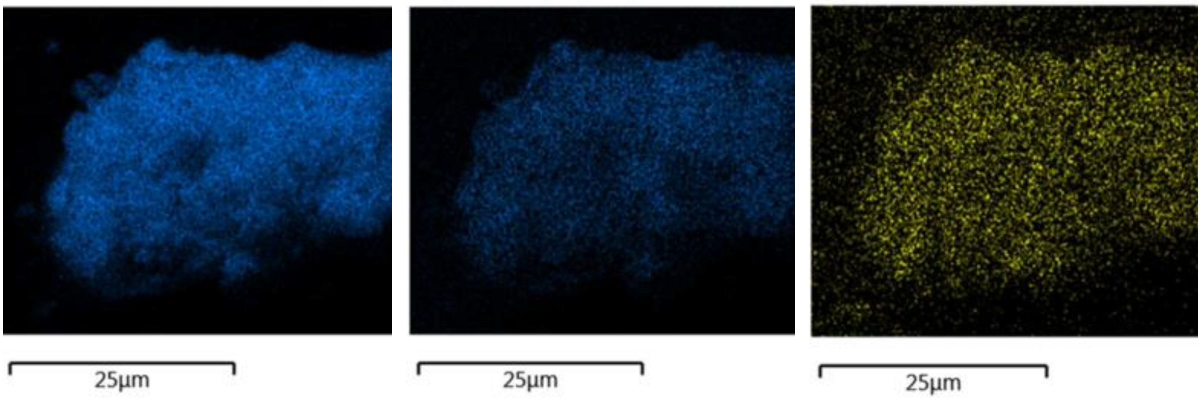

Si (K10) Mo In

HSiMo/K10

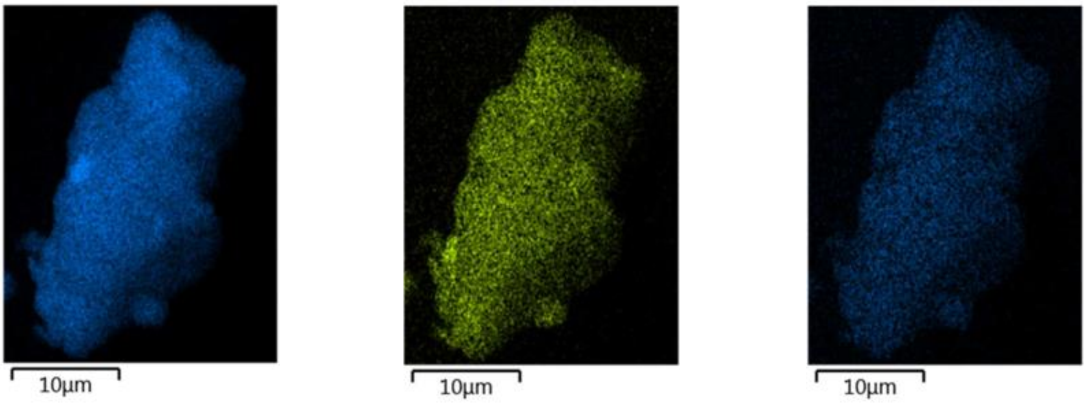

Si (K10 & HSiMo) Al (K10) Mo

Figure S 4: SEM EDX-Mapping of HPAs supported on K10

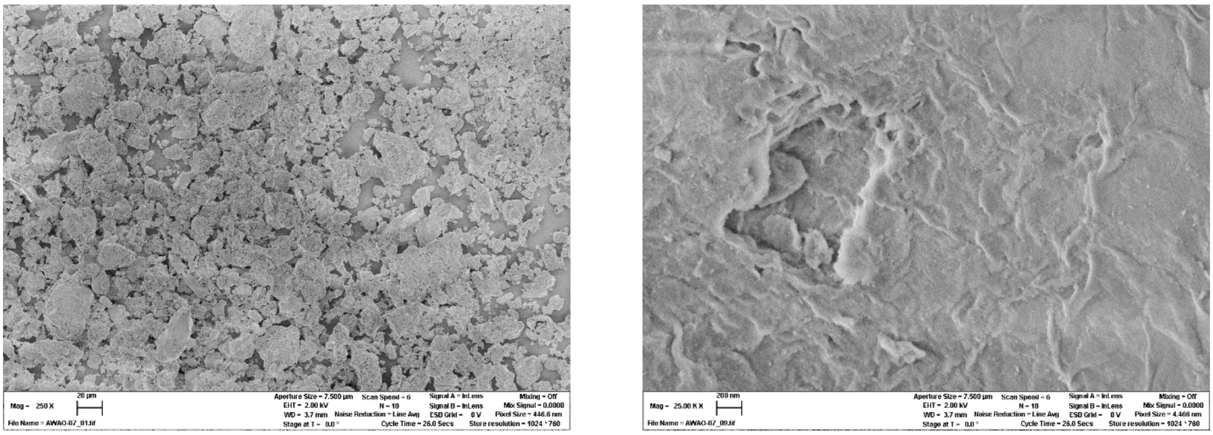

SEM images of HSiW/K10 at magnifications of 250x and 25,000x.

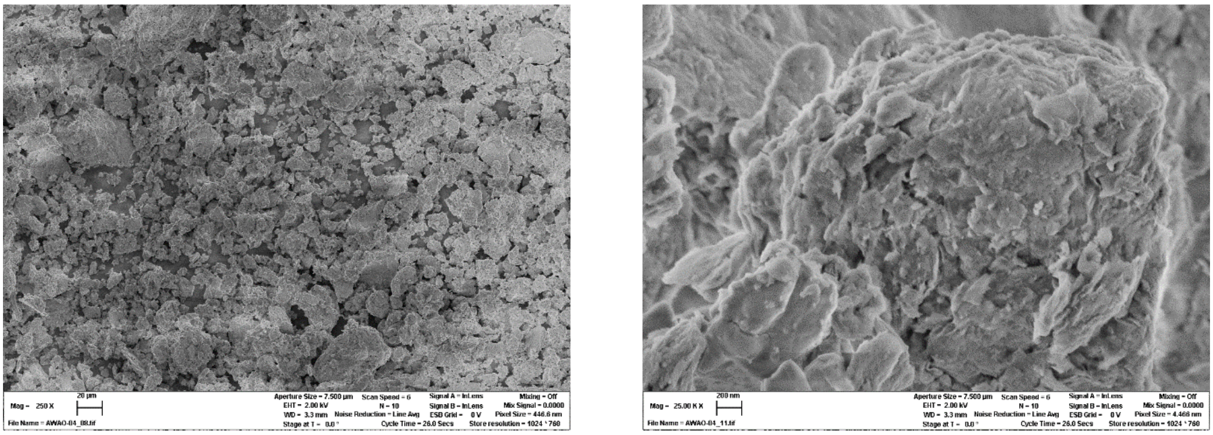

SEM images of HPMo/K10 at magnifications of 250x and 25,000x.

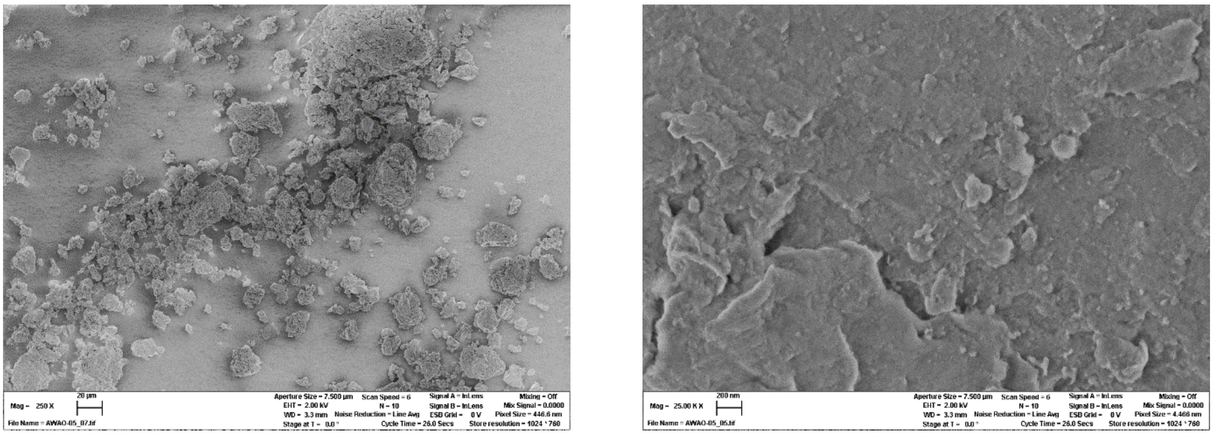

SEM images of HPW/K10 at magnifications of 250x and 25,000x.

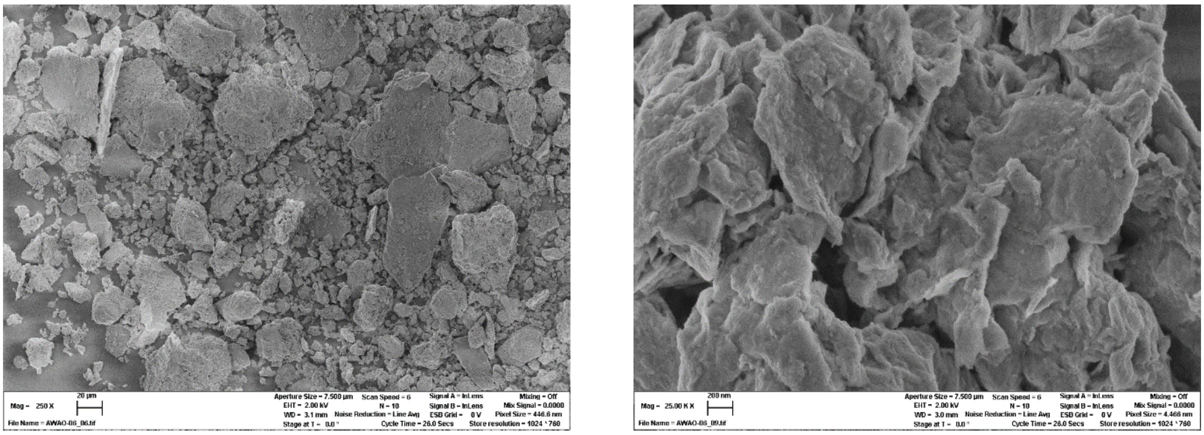

SEM images of HPVMo/K10 at magnifications of 250x and 25,000x.

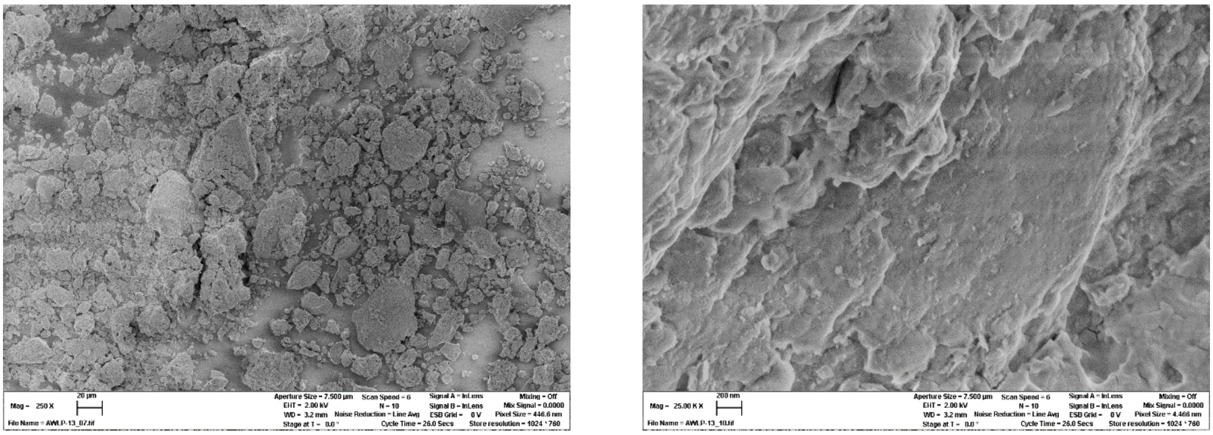

SEM images of HPInMo/K10 at magnifications of 250x and 25,000x.

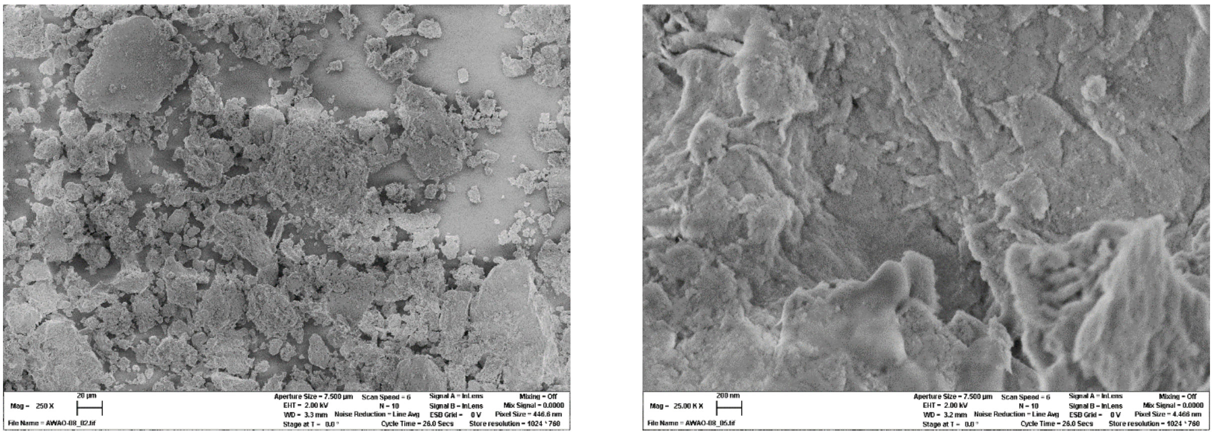

SEM images of HSiMo/K10 at magnifications of 250x and 25,000x.

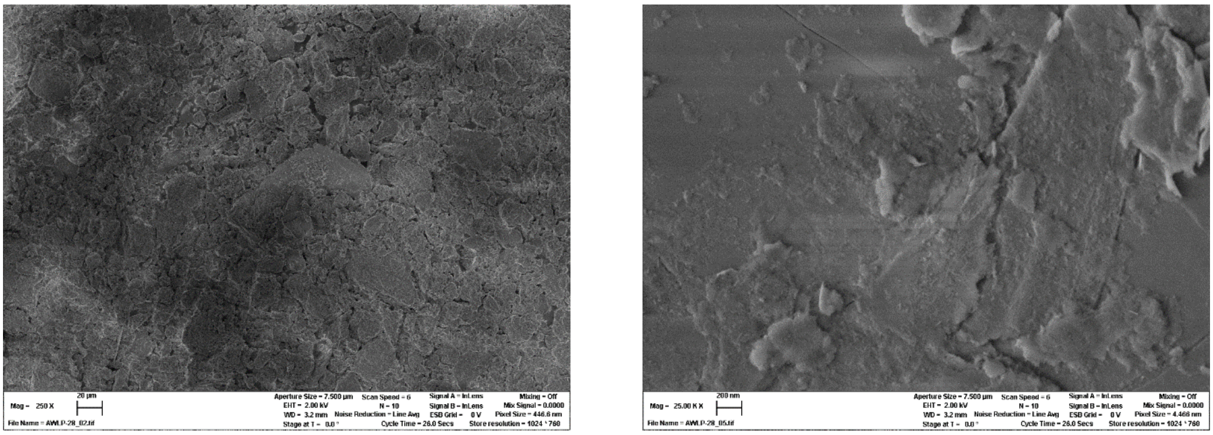

SEM images of K10 at magnifications of 250x and 25,000x.

Figure S 5: SEM Images of HPAs supported on K10.

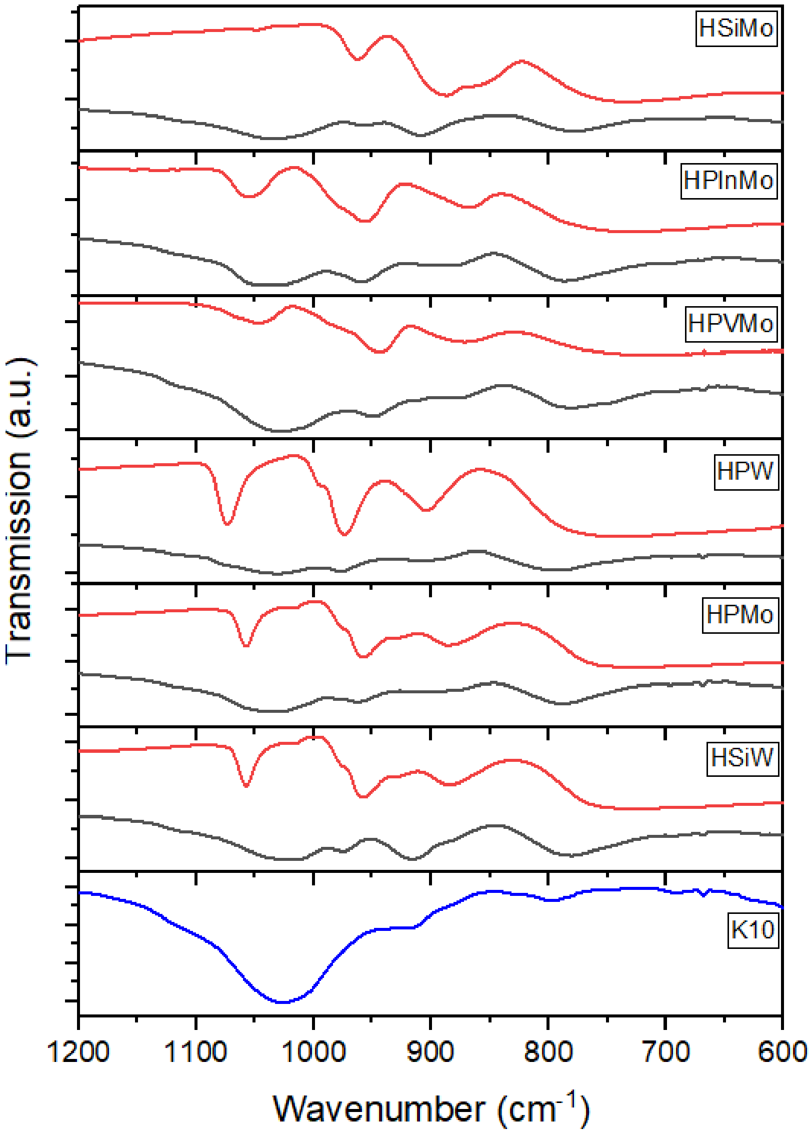

Figure S 6: IR spectra of pure (red line) and HPAs supported on K10 (black line) and pure K10 (blue).

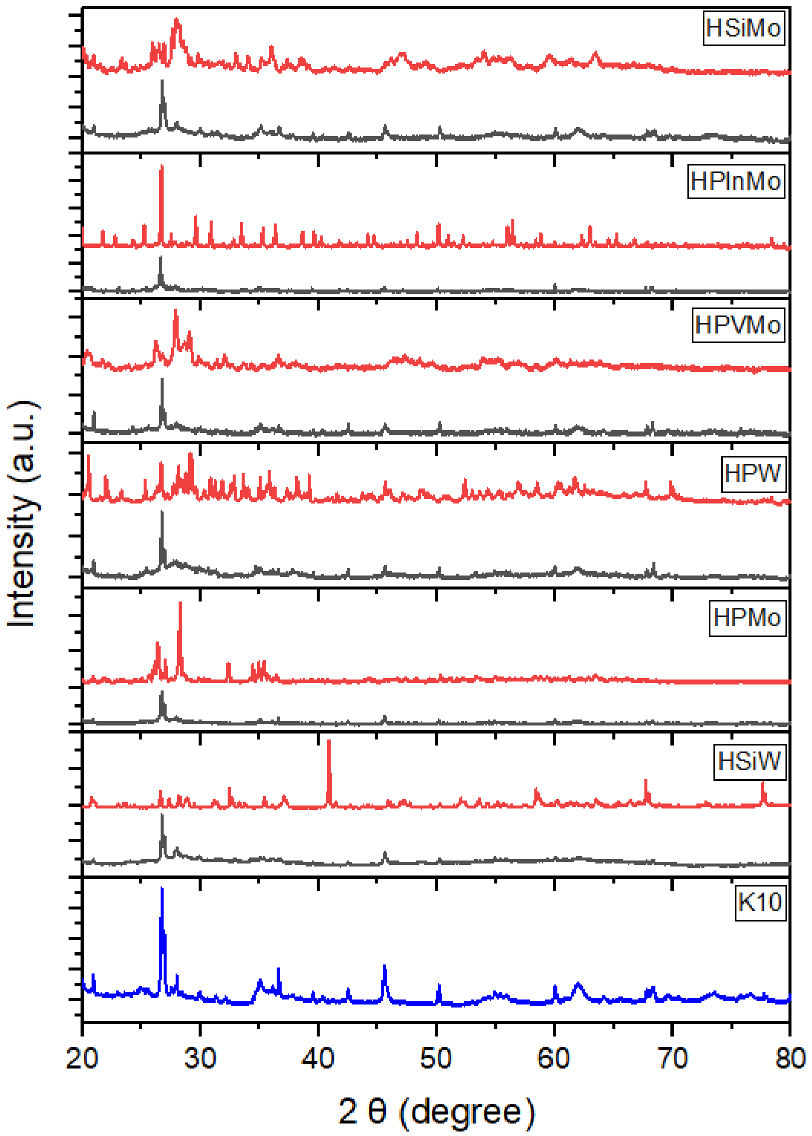

Figure S 7: XRD analytics of pure (red line) and HPAs supported on K10 (black line) and pure K10 (blue).

Table S 2: Catalytic results of HPAs supported on K10. Reaction conditions: T = 250 °C, p = 50 bar, H_2/CO_2 3/1, GHSV = 10000 h^{-1}.

catalyst	HSiW	HPMo	HPW	HPVMo	HPInMo	HSiMo	Pure K10
X_{CO_2} (%)	18.85	18.78	20.27	20.31	20.45.	19.96	19.89
Y_{MeOH} (%)	3.88	3.96	5.41	6.98	6.51	5.58	5.86
Y_{DME} (%)	7.06	7.10	5.73	3.95	4.69	5.24	4.76
Y_{CO} (%)	11.44	11.27	11.99	11.36	11.60	11.75	11.64
S_{MeOH} (%)	17.36	17.73	23.39	31.34	28.54	24.74	26.34
S_{DME} (%)	31.54	31.81	24.78	17.71	20.58	23.21	21.38
S_{CO} (%)	51.11	50.46	51.82	50.95	50.87	52.05	52.28
P_{mass} (g_{DME} g_{cat}^{-1} h^{-1})	0.48	0.48	0.39	0.27	0.32	0.36	0.32
P_{mol} (mol_{DME} mol_{HPA}^{-1} h^{-1})	77.84	59.40	55.53	31.12	31.00	41.30	-

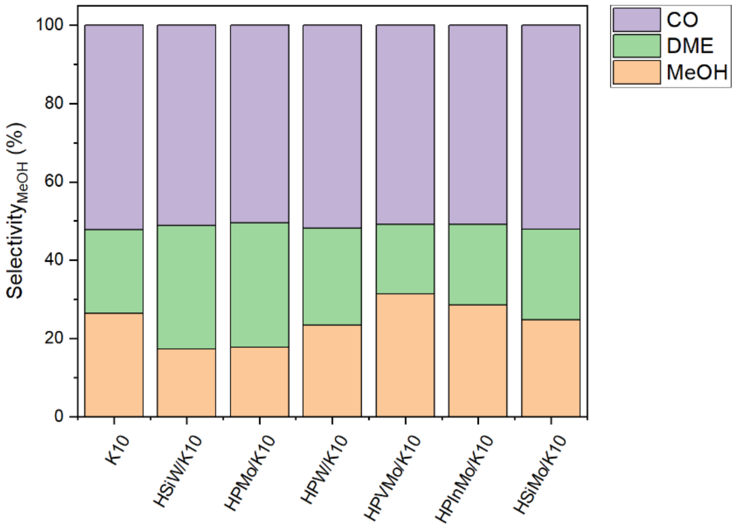

Figure S 8: Selectivities of HPAs supported on K10. Reaction conditions: T = 250 °C, p = 50 bar, H_2/CO_2 3/1, GHSV = 10000 h^{-1}.

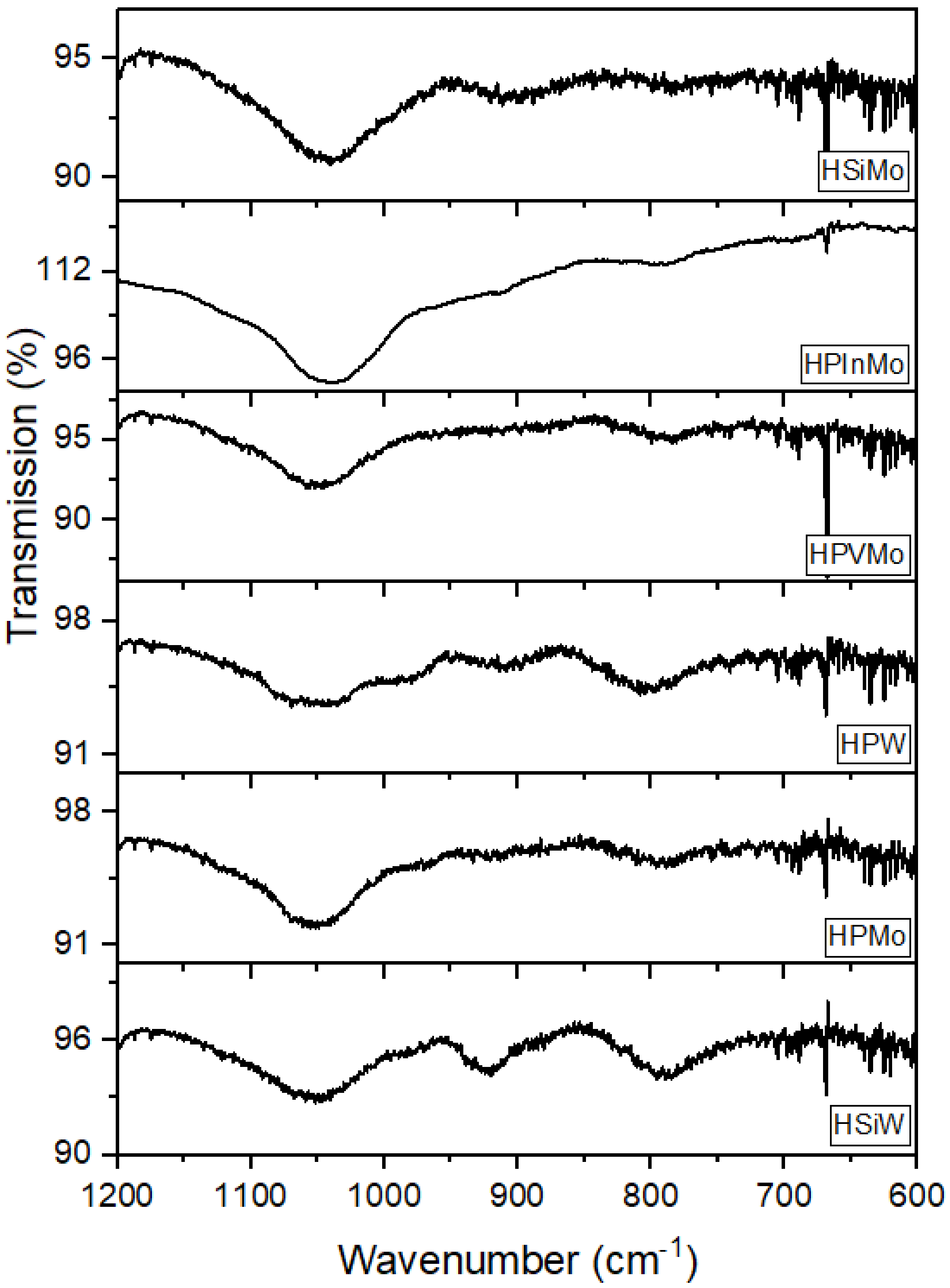

Figure S 9:IR analytics of HPAs supported on K10 after reaction.

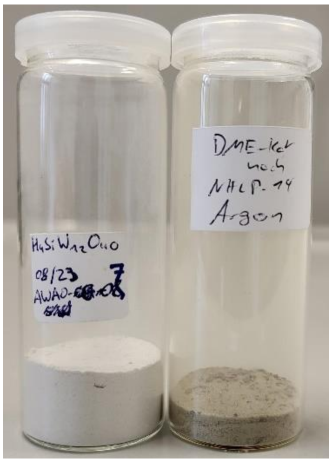

HSiW/K10

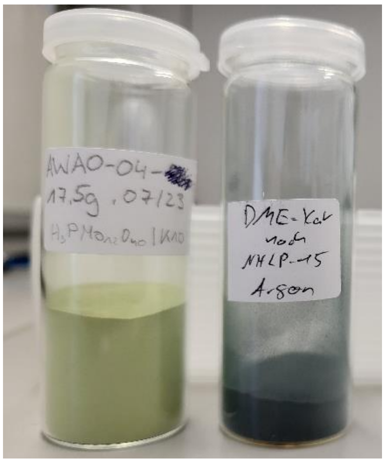

HPMo/K10

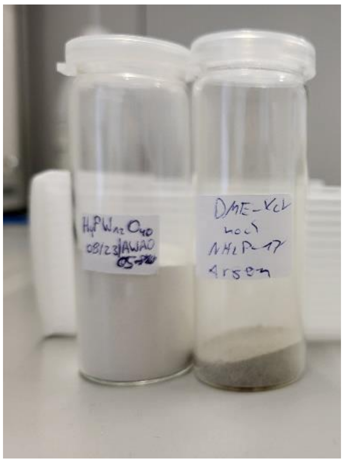

HPW/K10

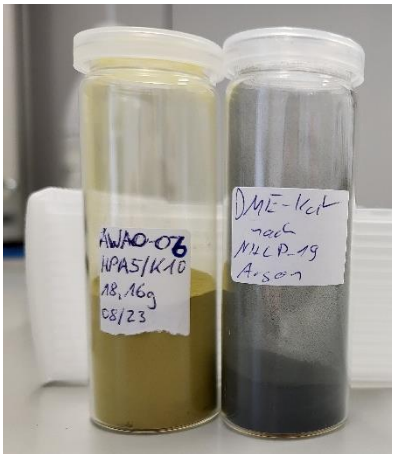

HPVMo/K10

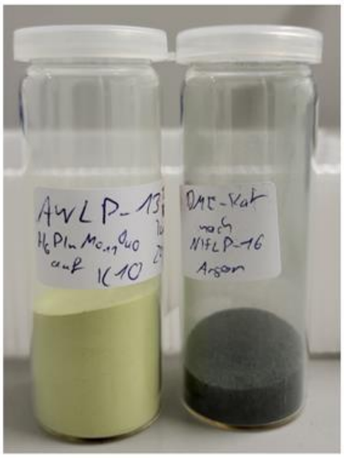

HPInMo/K10

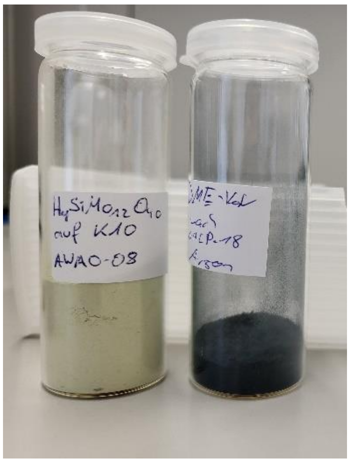

HSiMo/K10

Figure S 10: HPAs supported on K10 before (left) and after use (right) in DME synthesis.

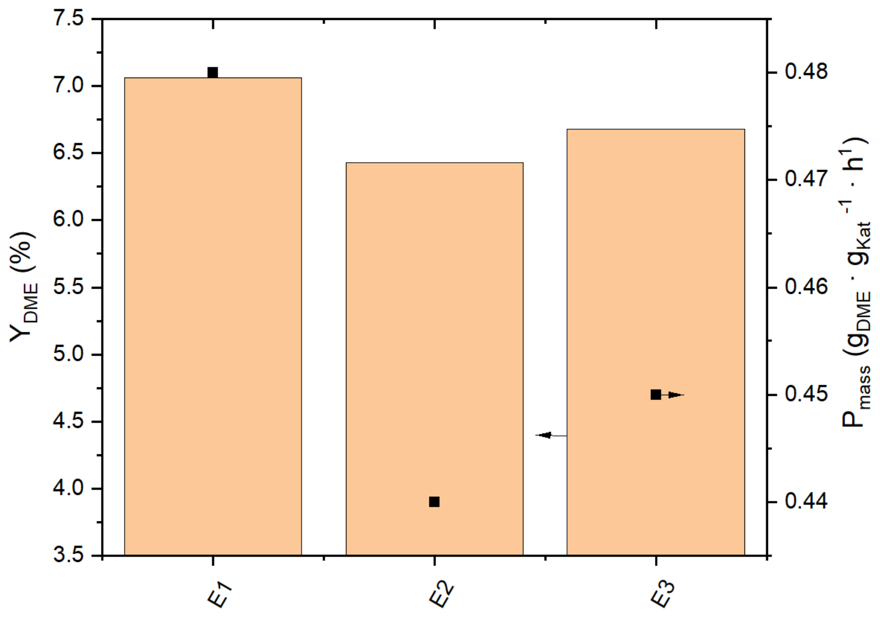

Figure S 11: Yield of DME Y_{DME} and productivity P_{mass} of reproduction experiments E1-E3 with HSiW/K10. Reaction conditions: T = 250 °C, p = 50 bar, H_2/CO_2 3/1, GHSV = 10000 h^{-1}.

Table S 3: *Catalytic results of reproduction experiments using $Cu/ZnO/Al_2O_3$ and HSiW/K10 as catalysts. Reaction conditions: T = 250 °C, p = 50 bar, H_2/CO_2 3/1, GHSV = 10000 h^{-1}.*

	E1	E2	E3	E4*	Mean value (E1-E3)	Standard deviation (E1-E3)
X_{CO_2} (%)	18.85	19.96	19.92	20.72	19.58	0.63
Y_{MeOH} (%)	3.88	4.79	4.31	6.38	4.33	0.46
Y_{DME} (%)	7.06	6.43	6.68	5.38	6.72	0.32
Y_{CO} (%)	11.44	11.96	12.27	11.65	11.89	0.42
S_{MeOH} (%)	17.36	20.65	18.54	27.24	18.85	1.67
S_{DME} (%)	31.54	27.73	28.72	22.99	29.33	1.98
S_{CO} (%)	51.11	51.62	52.74	49.77	51.82	0.83
P_{mass} (g_{DME} g_{cat}^{-1} h^{-1})	0.48	0.44	0.45	0.37	0.46	0.02

*In reproduction experiment E4, a reduced DME yield of 5.38% is noted, likely due to a measurement error, as the CO_2 conversion and CO yield are consistent with the other experiments, suggesting proper catalyst packing. Potential errors, such as incorrect DME catalyst layer packing or gas flow rate settings to the gas chromatograph, might contribute to lower detected DME levels. Consequently, E4 was excluded from the mean and standard deviation calculations to ensure accuracy.

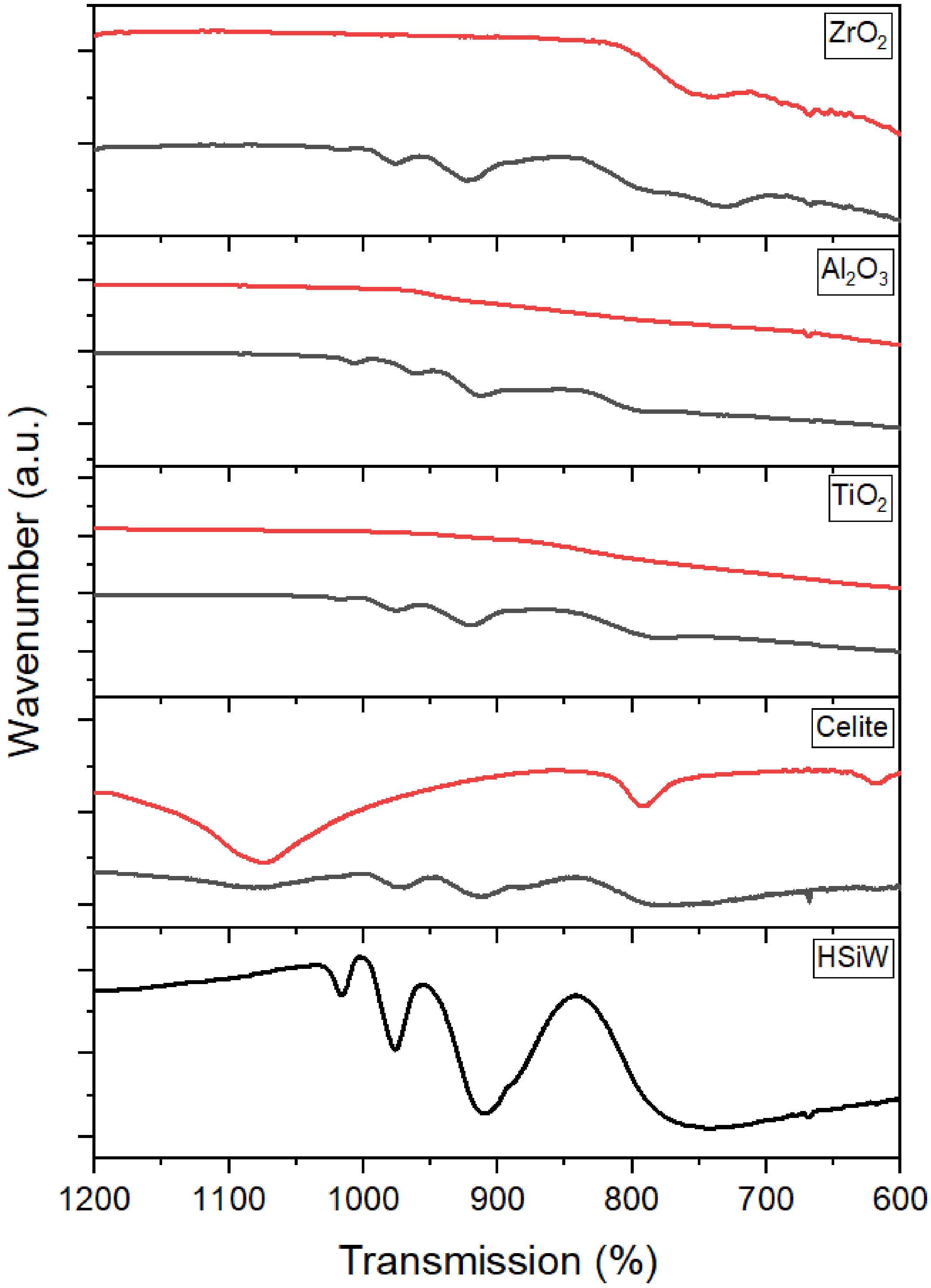

Figure S 12: IR analytics of pure supports (red line) and supported HSiW (black line).

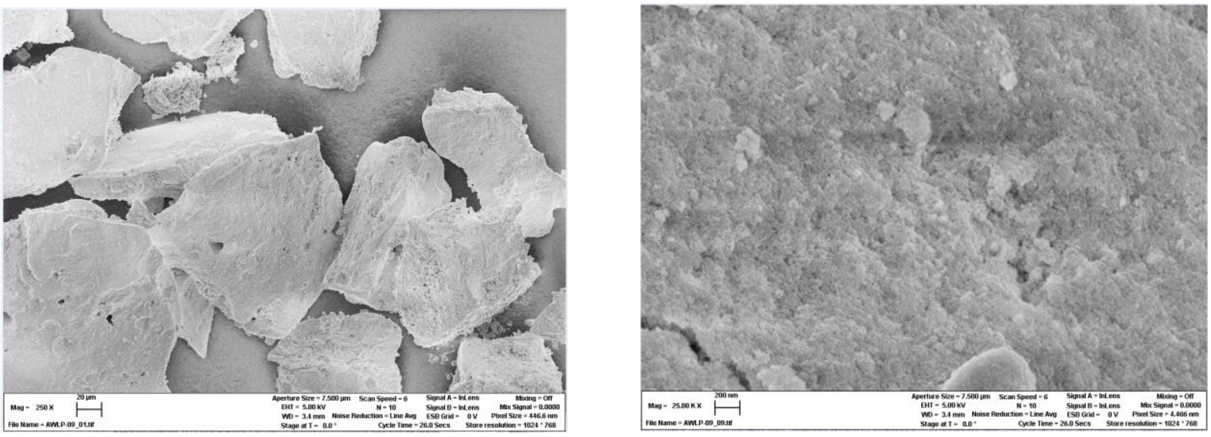

SEM images of $HSiW/ZrO_2$ at magnifications of 250x and 25,000x.

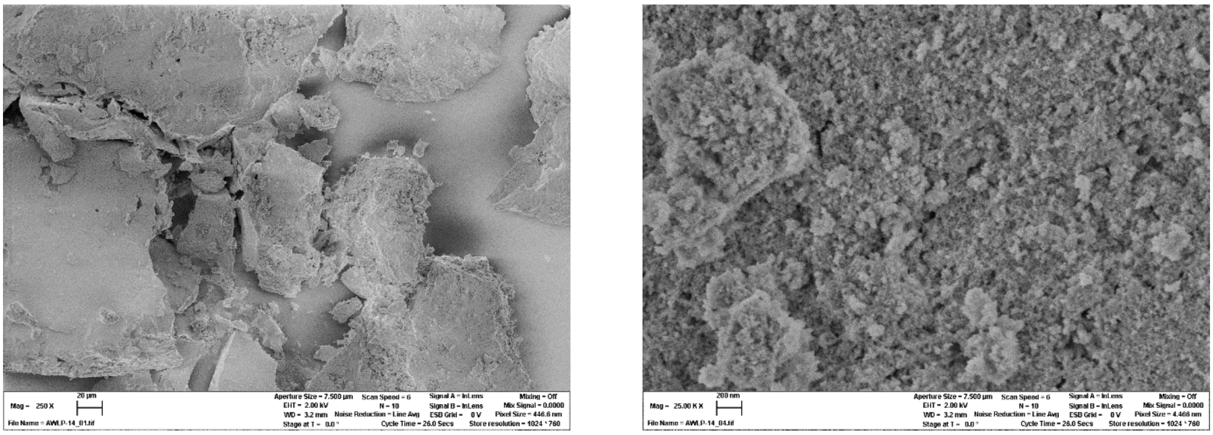

SEM images of ZrO_2 at magnifications of 250x and 25,000x.

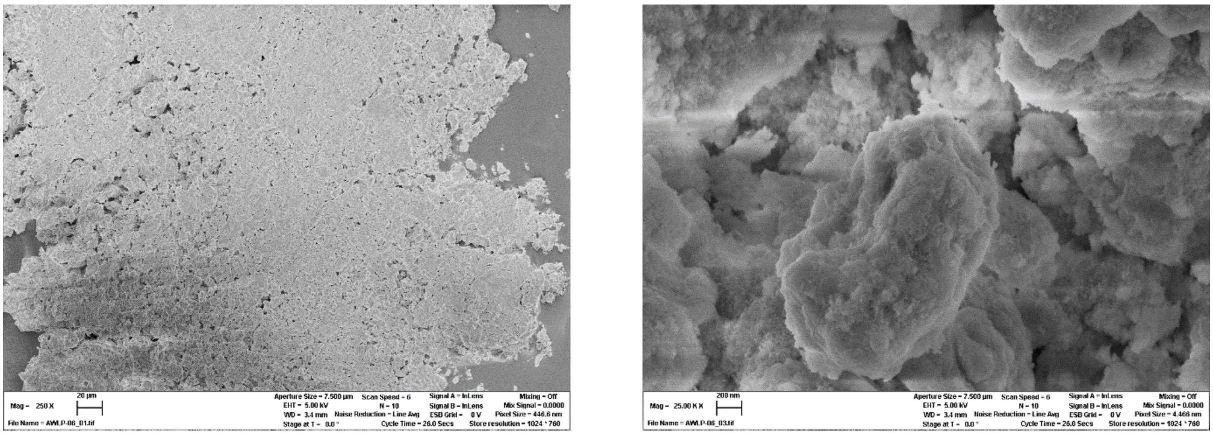

SEM images of $HSiW/Al_2O_3$ at magnifications of 250x and 25,000x.

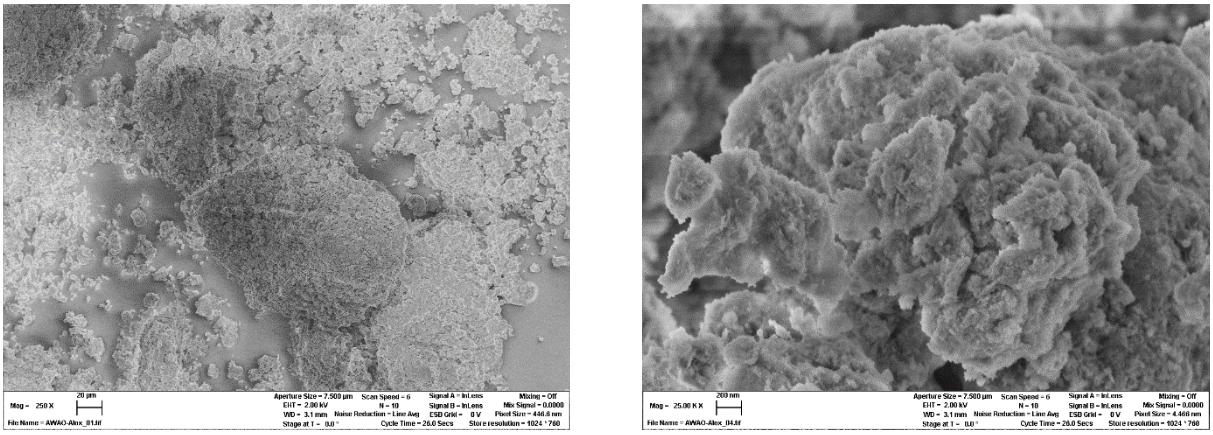

SEM images of Al_2O_3 at magnifications of 250x and 25,000x.

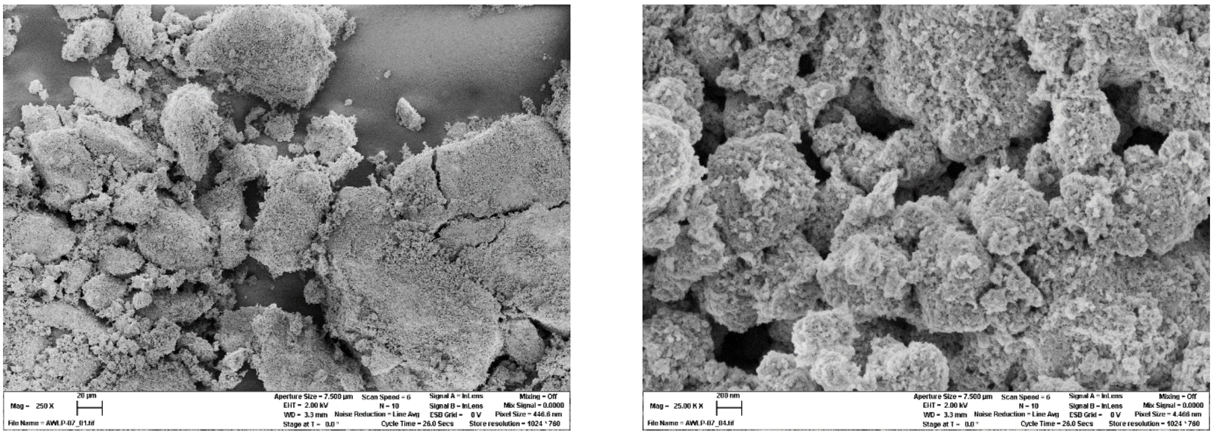

SEM images of HSiW/TiO_2 at magnifications of 250x and 25,000x.

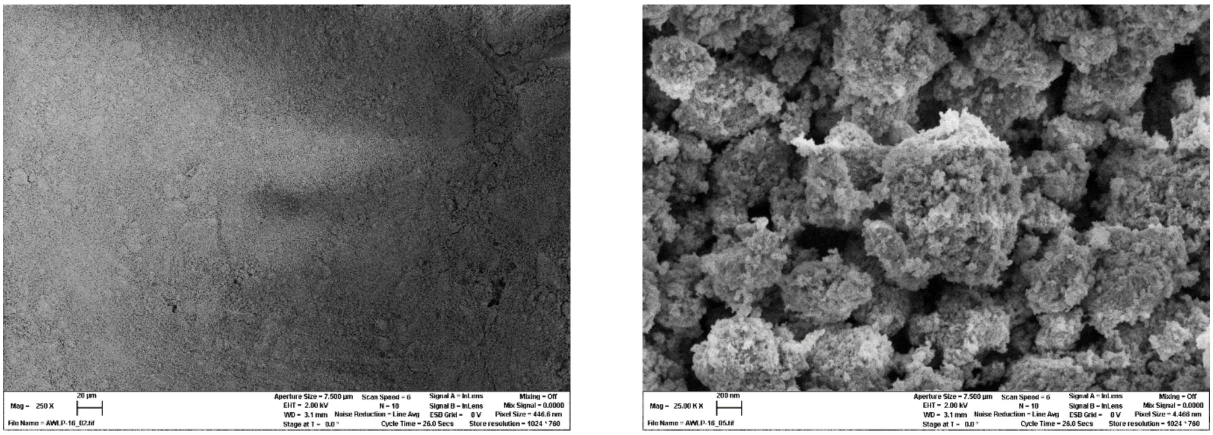

SEM images of TiO_2 at magnifications of 250x and 25,000x.

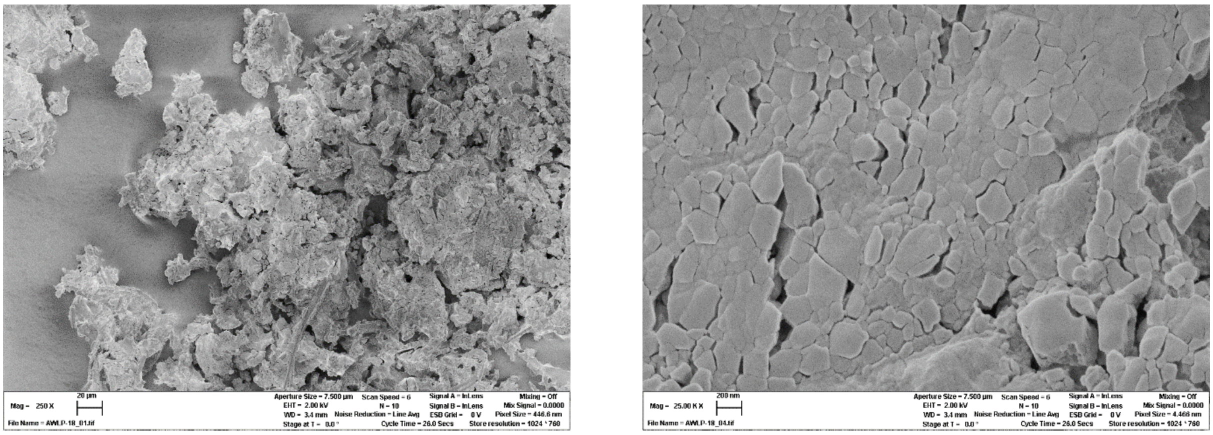

SEM images of HSiW/Celite at magnifications of 250x and 25,000x.

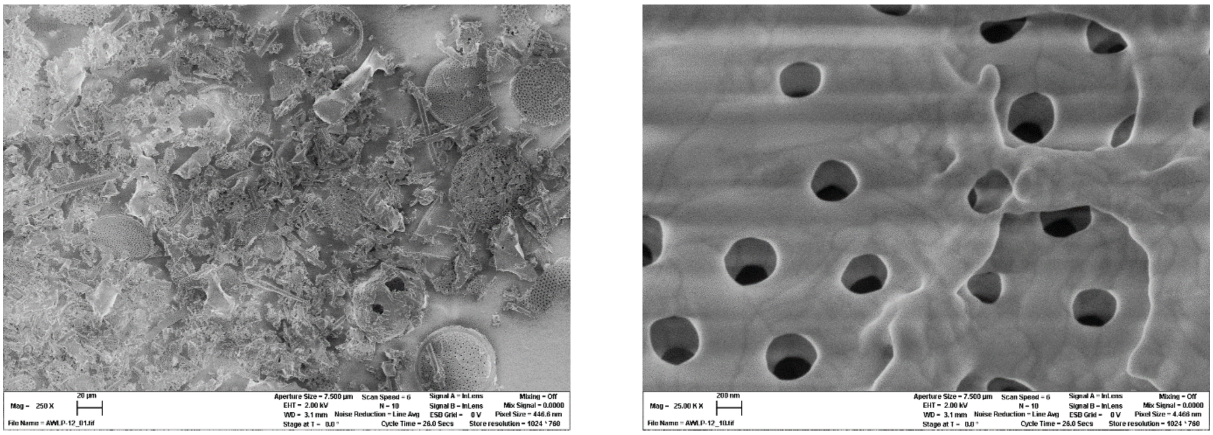

SEM images of Celite at magnifications of 250x and 25,000x.

Figure S 13: SEM Images of HSiW on different supports.

HSiW/ZrO_2

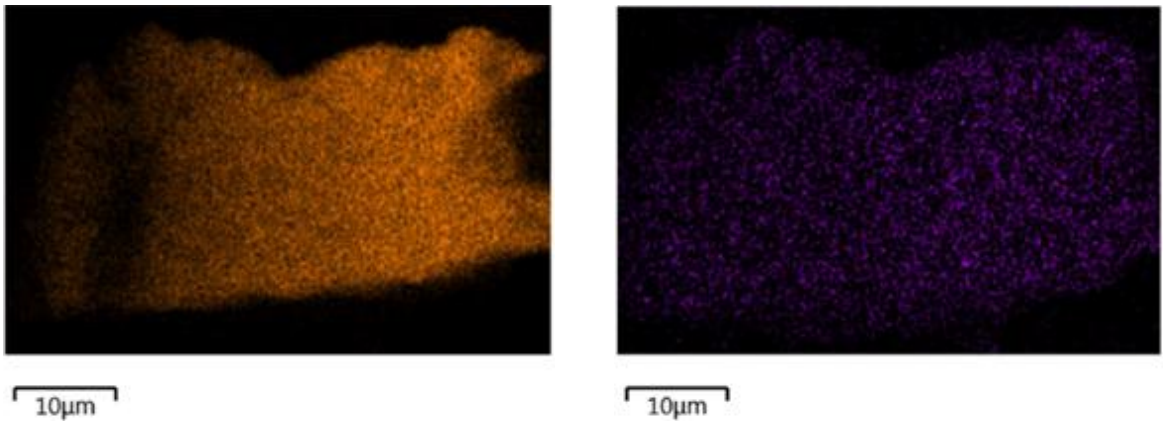

Zr W

HSiW/Al_2O_3

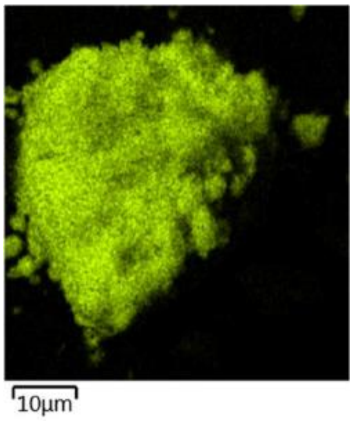

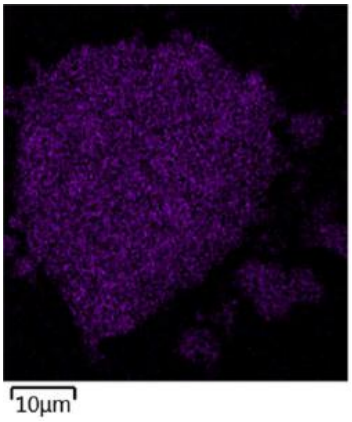

Al W

HSiW/TiO_2

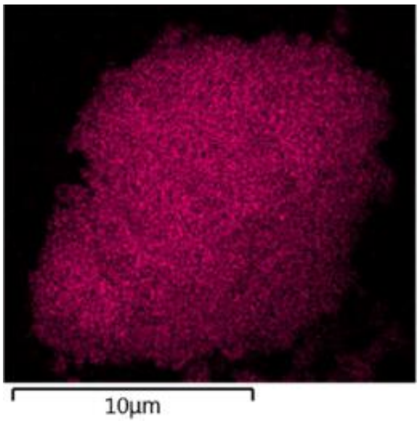

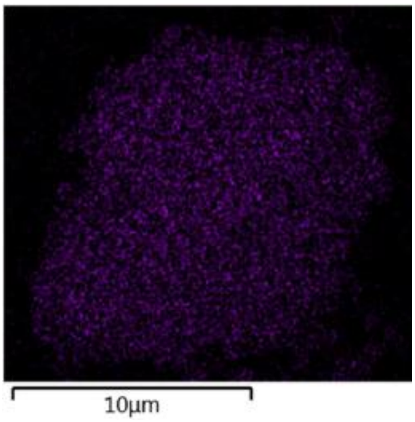

Ti W

HSiW/Celite

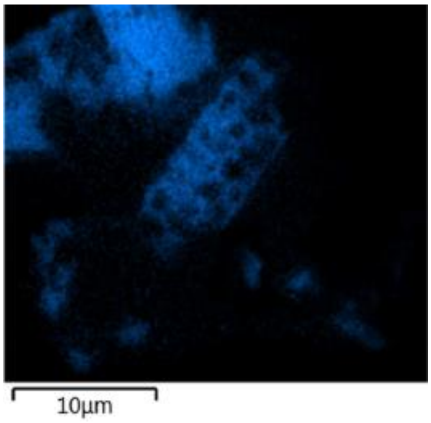

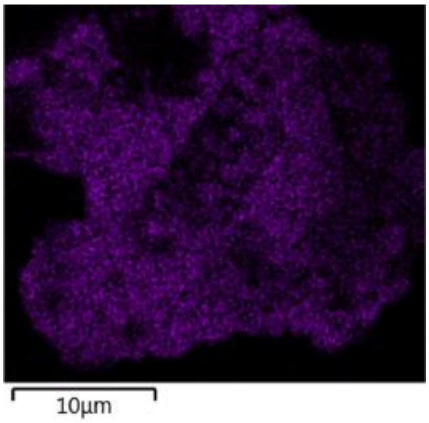

W

Figure S 14: SEM EDX-Mapping of HSiW on different supports.

Table S 4: Catalytic results of HSiW on different supports. Reaction conditions: T = 250 °C, p = 50 bar, H_2/CO_2 3/1, GHSV = 10000 h^{-1}.

catalyst	HSiW/ ZrO_2	ZrO_2	HSiW/ Al_2O_3	Al_2O_3	HSiW/ TiO_2	TiO_2	HSiW/ Celite	Celite
X_{CO_2} (%)	19.36	24.43	20.39	24.43	18.98	23.76	18.65	23.99
Y_{MeOH} (%)	3.32	12.75	4.23	12.34	3.35	12.41	3.51	13.18
Y_{DME} (%)	7.08	0.00	6.69	0.10	6.92	0.02	6.81	0.00
Y_{CO} (%)	12.50	11.68	12.82	12.03	12.17	11.35	11.74	10.81
S_{MeOH} (%)	14.50	52.19	17.80	50.42	14.95	52.18	15.93	54.95
S_{DME} (%)	30.91	0.00	28.17	0.42	30.84	0.10	30.86	0.00
S_{CO} (%)	54.59	47.81	54.02	49.17	54.21	47.72	53.21	45.05
P_{mass} ($g_{DME}\ g_{cat}^{-1}\ h^{-1}$)	0.48	0.00	0.45	0.01	0.47	0.00	0.46	0.00
P_{mol} ($mol_{DME}\ mol_{HPA}^{-1}\ h^{-1}$)	125.44	-	65.39	-	77.74	-	47.68	-

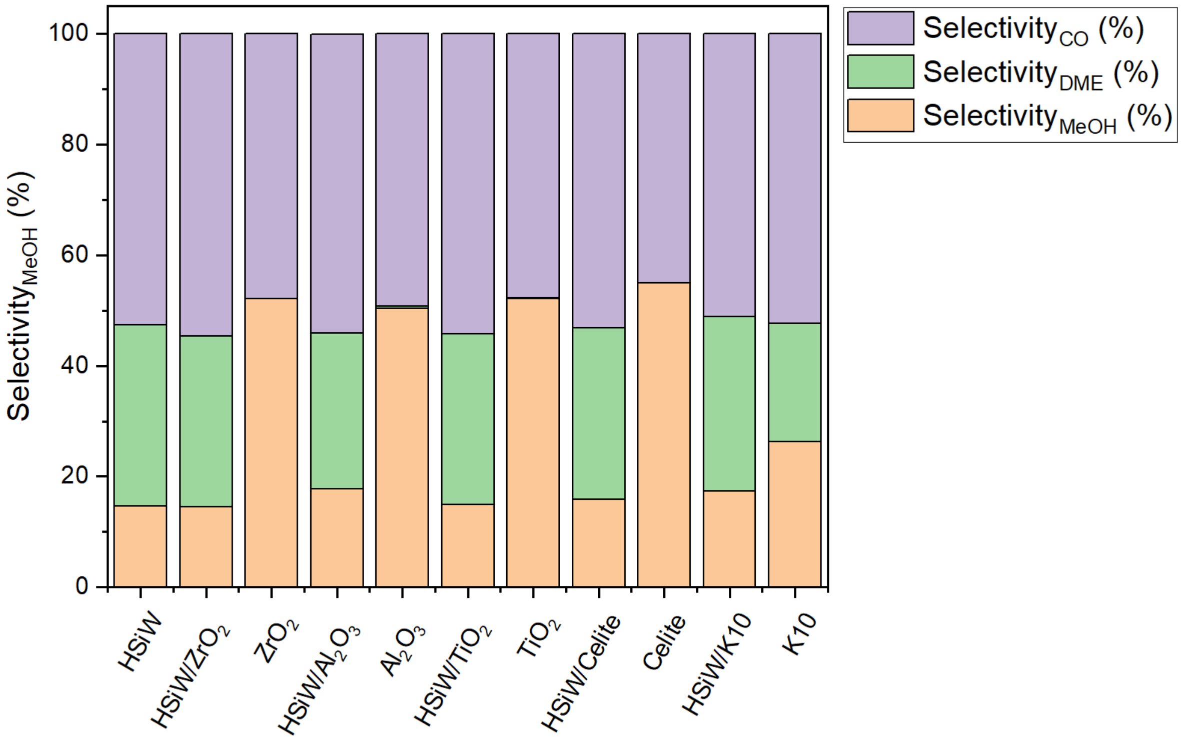

Figure S 15: Selectivities of HSiW on different supports. Reaction conditions: T = 250 °C, p = 50 bar, H_2/CO_2 3/1, GHSV = 10000 h^{-1}.

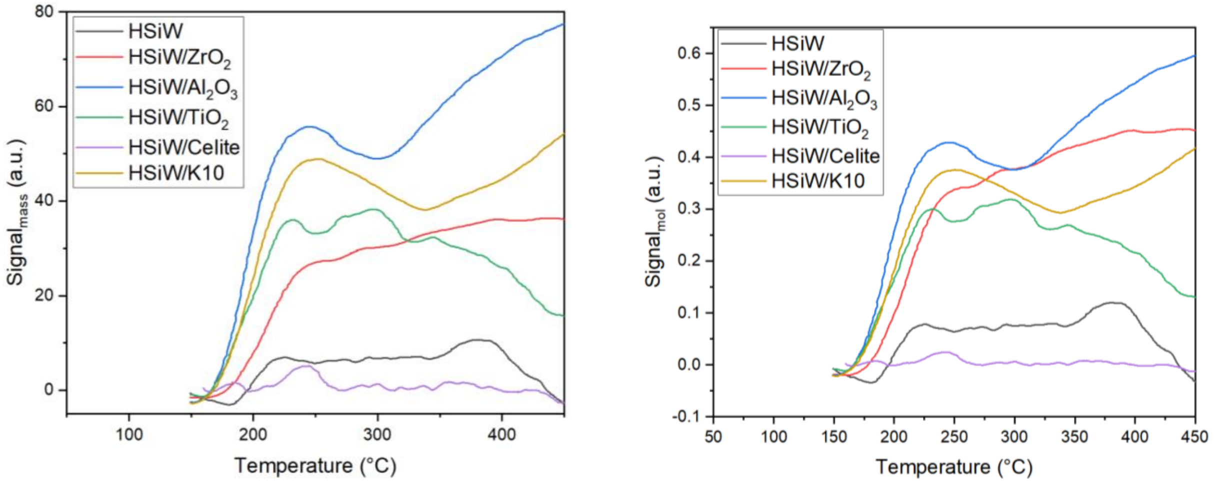

Figure S 16: NH_3-TPD analysis of HSiW on different supports, normalized to mass of catalyst (left) and normalized to molar mass of supported HPA (right).

Comparative Analysis with Previously-Reported Catalyst

The IR spectrum of HSiW/ZrO_2K exhibits characteristic Keggin vibration bands, confirming the preservation of the Keggin structure after impregnation (Figure S17). Elemental analysis confirms a slightly higher loading for HSiW/ZrO_2^K compared to HSiW/ZrO_2^W. N_2-physisorption further indicates similar surface areas, with marginally smaller pore diameters and pore volumes for HSiW/ZrO_2K (Table S5). Unlike the wet impregnation method used in this study, the modified synthesis procedure by Kubas utilized ethanol instead of water and reduced impregnation time, potentially leading to increased deposition and thus higher POM loading on the support material ZrO_2, as well as the slightly reduced pore volumes and diameters. However, these results should be interpreted cautiously, as the variations in elemental analysis and N_2 physisorption measurements are within the margin of error for both methods.

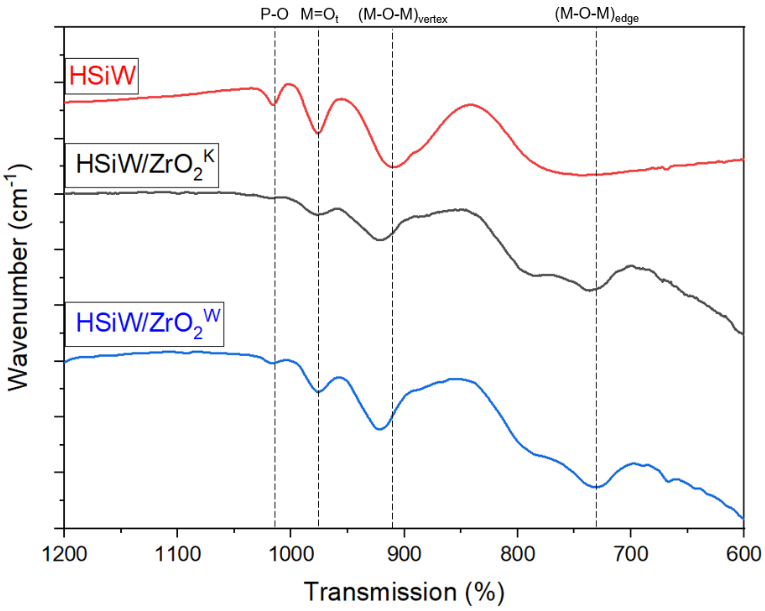

Figure S 17: IR spectra for HPA/ZrO_2 of current study (HPA/ZrO_2W) vs. catalyst from literature (HPA/ZrO_2K) and pure HiSW.

Table S 5: Textural properties and results of elemental analysis for HPA/ZrO_2 of current study (HPA/ZrO_2W) vs. catalyst from literature (HPA/ZrO_2K).

	$HSiW/ZrO_2^W$	$HSiW/ZrO_2^K$
Textural properties		
S_{BET} (m^2/g)	81	80
Ø pore diameter (nm)	3.40	3.38
Pore volume (mL/g)	0.18	0.11
Elemental analysis		
W (wt.%)	18.32	20.55
HPA (wt.%)	27.19	30.49
$Loading_{eff.}$ ($\mu mol_{HPA}\ g_{cat}^{-1}$)	80	90
$Loading_{theor.}$ ($\mu mol_{HPA}\ g_{cat}^{-1}$)	90	90

References

(1) Odyakov, V. F.; Zhizhina, E. G. A novel method of the synthesis of molybdovanadophosphoric heteropoly acid solutions, *React Kinet Catal Lett.* **2008**, *95,* pp. 21–28.

(2) Raabe, J.-C.; Poller, M. J.; Voß, D.; Albert, J. H8 PV5 Mo7 O40 - A Unique Polyoxometalate for Acid and RedOx Catalysis: Synthesis, Characterization, and Modern Applications in Green Chemical Processes, *ChemSusChem.* **2023**, *16,* e202300072.

(3) Raabe, J.-C.; Aceituno Cruz, J.; Albert, J.; Poller, M. J. Comparative Spectroscopic and Electrochemical Study of V(V)-Substituted Keggin-Type Phosphomolybdates and -Tungstates, *Inorganics.* **2023**, *11,* p. 138.

(4) Strickland, J. D. H. The Preparation and Properties of Silicomolybdic Acid. I. The Properties of Alpha Silicomolybdic Acid, *J. Am. Chem. Soc.* **1952**, *74,* pp. 862–867.

(5) Wesner, A.; Kampe, P.; Herrmann, N.; Eller, S.; Ruhmlieb, C.; Albert, J. Indium-based Catalysts for CO 2 Hydrogenation to Methanol: Key Aspects for Catalytic Performance, *ChemCatChem.* **2023**, *15*.
